# Emergence of Shared Intentionality Is Coupled to the Advance of Cumulative Culture

**DOI:** 10.1371/journal.pcbi.1004587

**Published:** 2015-10-30

**Authors:** Simon D. Angus, Jonathan Newton

**Affiliations:** 1 Department of Economics, Monash University, Victoria, Australia; 2 School of Economics, University of Sydney, New South Wales, Australia; University of Chicago, UNITED STATES

## Abstract

There is evidence that the sharing of intentions was an important factor in the evolution of humans’ unique cognitive abilities. Here, for the first time, we formally model the coevolution of jointly intentional behavior and cumulative culture, showing that rapid techno-cultural advance goes hand in hand with the emergence of the ability to participate in jointly intentional behavior. Conversely, in the absence of opportunities for significant techno-cultural improvement, the ability to undertake jointly intentional behavior is selected against. Thus, we provide a unified mechanism for the suppression or emergence of shared intentions and collaborative behavior in humans, as well as a potential cause of inter-species diversity in the prevalence of such behavior.

## Author Summary

A typical day in the life of almost any person involves the sharing of intentions. Such shared intentions range from the banal—‘we intend to meet for dinner’, to the elegant—‘we intend to sing a duet’, to those with far reaching consequences—‘we intend to form an alliance to defeat our mutual enemy’. Recent research in developmental psychology suggests that humans’ especial proclivity to undertake jointly intentional behavior could be responsible for the uniqueness of human cognition. That is, humans do not only collaborate because we are smart, but are smart because we collaborate. Using recent advances in game theoretic modeling, we, for the first time, formally model the evolution of the ability to form shared intentions and show that this ability is likely to have evolved at a time when technological and cultural progress offered particularly high benefits to survival, such as might be the case during a period of significant environmental change.

## Introduction

It has been hypothesized that the evolution of modern human cognition was catalyzed by the development of jointly intentional modes of behavior. That is, a high degree of collaborative behavior amongst early hominins relative to other great apes aided the development of the rich cognitive abilities found in modern humans. This is known as as the shared intentionality hypothesis [1] or the Vygotskian intelligence hypothesis [2–4]. Considerable experimental evidence suggests that from an early age (1–2 years), human infants outperform apes at tasks that involve collaborative activity [5, 6]. Specifically, human infants excel at joint action motivated by reasoning of the form “we intend to do X” (shared intentions [7–9]), as opposed to reasoning of the form “I intend to do X [because he is doing X]” (individual intentions).

Jointly intentional action pervades human existence, from the mundanity of you and your partner choosing a color of wallpaper for your house to the exquisite plays of your favorite sports team. Examples from the philosophy literature include painting a house [9], pushing a car [8], and even going for a walk together with another person [10]. Even in economics, the most individualistic of the social sciences, concepts that embody shared intentions, such as the ‘firm’ and the ‘household’, are routinely used as units of agency.

Just as rational choice makes individual intentions subject to optimality constraints at the individual level, logic suggests that shared intentions should be subject to optimality constraints at the collective level: if *we* are forming an intention, it should not be Pareto inferior to some other intention that we could form. It is relatively simple to incorporate such logic into dynamic models of behavior and there exists a recent literature in evolutionary game theory that does this [11–14]. However, the question of how and why this most ubiquitous of human traits—the ability to undertake jointly intentional behavior—would have emerged, is an open one for which no formal models currently exist, although it is natural to conjecture that an environment rich in coordination problems would be highly conducive to its emergence [15, 16].

Here we formally model the evolution of jointly intentional action and show under what conditions it is likely to emerge. Using a multi-level selection framework similar to [17, 18], we model competition between different bands of individuals, called *demes*. Within each deme, interactions of hunter-gatherers are modeled as coordination games [19] on a social network. The demes are engaged in a process of *cultural accumulation*: step by step, demes move up a technology ladder, gradually adopting new and better technologies. Within any given deme, the process by which a new technological norm takes over from an old one is familiar to the networks literature [20–25]. However, unlike these previous models, any given individual within a deme may be either of two types. *N type* individuals lack the ability to share intentions. Their behavior is individualistic and determined in a similar manner to the papers just cited. *SI type* individuals have the ability to share intentions and can adjust their choice of strategy in tandem with any of their neighbors to their mutual benefit.

The main results of the paper follow from the effects of jointly intentional behavior on the speed of convergence to improved technological norms. The insight we derive, by explicitly modeling techno-cultural advance, is to link technology adoption to the evolution of collaboration. The collaborative sharing of intentions can either speed or slow the adoption of new norms on a social network [14]. If the benefits from adopting new technologies or norms are low but positive, then widespread sharing of intentions within a deme slows the adoption of new technologies. These demes fall behind other demes and are selected against at an inter-demic level. Thus the collaborative sharing of intentions is selected against. Conversely, if the benefits from adopting new technologies or norms are high, such as may be the case during a period of rapid environmental change [26, 27], then widespread sharing of intentions within a deme speeds the adoption of new technologies. These demes gain a technological advantage over other demes and succeed at inter-demic competition. Shared intentionality evolves and rapidly becomes dominant in the population. This emergence of shared intentions at a time of significant technological advance is consistent with evidence that composite tool manufacture may have evolved roughly contemporaneously (∼ 300 ka) with grammatical language [28], the primary application of which is the facilitation of jointly intentional behavior. This concords with the ‘cultural intelligence hypothesis’ which suggests that socially learned culture has affected the evolution of cognitive traits [29, 30].

To the best of the authors’ knowledge, this is the first paper to formally model the evolution of the ability to undertake jointly intentional behavior, although the work of Bacharach [31] makes tentative steps in this direction. Collaborative sharing of intentions directly alters *how* strategies are chosen, not *which* strategies are chosen, and although the former affects the latter, how it does so depends on circumstances. Pairs of individuals can and do collaborate to coordinate on both innovative and status quo technologies. This behavior is *mutualistic*, not altruistic or spiteful [32]. That is to say, when a pair of individuals undertakes jointly intentional activity, they *both* gain from doing so. Moreover, we look at coordination games, not prisoner’s dilemmas, so there are no gains to be made from cheating. Hence, it may be considered remarkable that the ability to collaboratively share intentions can be strictly selected against. The reason that it can be selected against is that collaboration aids coordination at a local level, which in some circumstances can hinder the spread of innovative behavior across a social network. That is, the presence of collective agency at a pairwise or small group level may be detrimental to the progress of larger society. Thus a methodological implication of our model is that adaptive dynamics of norm selection driven by a behavioral rule [21, 33], together with the evolution of a trait that affects the behavioral rule, lead to interesting results, even when the trait in question is mutualistic.

The mutualistic nature of the collective optimizing behavior of the current paper places it firmly outside of the game theoretic literature on the evolution of cooperation. This latter literature studies how individuals come to play some non-individually optimal ‘cooperative’ action. There is no such action in the current study and no individual does anything other than optimize. This is the reason we are careful to refer to the behavior we study as *collaborative* rather than *cooperative*. Finally, we mention in passing that our agency-based concept of collaboration is very different to [34], where ‘collaborative ability’ directly affects fitness via a production function.

## Model

**Fig 1:**
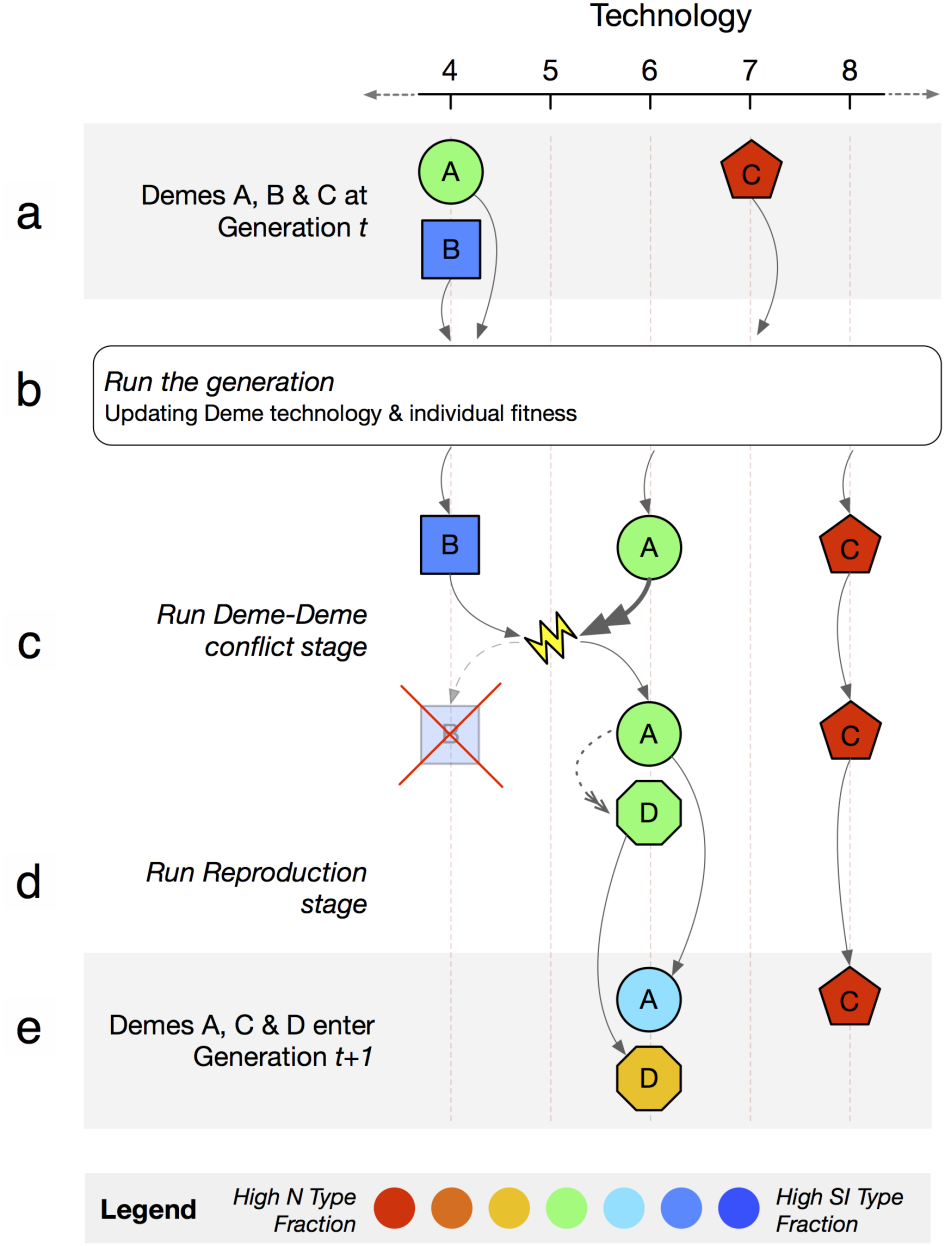
Model timing. **a:** Demes begin a generation with given technology and number of SI types. **b:** Interaction during a generation gives individual fitnesses and causes advances in deme technology (here, demes A and C increase their tech level). **c:** Some demes (here, deme B) face invasion by other demes (deme A). If the invading deme has higher technology, the invaded deme is eliminated and replaced by a replica of the invading deme (here, deme B is eliminated and replaced by deme D, a replica of deme A). **d:** Demes reproduce and populate the next generation via a finite population replicator dynamic (here, we see within-deme selection and genetic drift in demes A and D changing the number of SI types). **e:** Technology levels and number of SI types are carried forward into the next generation.

We summarize here the model. Details can be found in S1 Supporting Information. Following [17, 18], consider a metapopulation comprising *m* = 64 partially isolated subpopulations (called demes) of size *n* = 32 individuals. Each individual either has the ability to share intentions (type SI) or does not (type N). Time is divided into generations, each of which comprises *T* = 2000 periods. Individuals live for a single generation. Consider a given deme. At the start of a generation, the deme has achieved a level of technological/cultural sophistication *τ*. This will change as time passes, as will the share of SI and N types in the populations. That is, the model (summarized in Fig 1) is one of gene-culture coevolution.

### Within-deme interaction

The interaction structure within a deme is given by an undirected graph on the set of individuals in the deme. This is determined at the start of each generation, the idea being that for each individual there are a few individuals (the mean number, *d*, ranging from 4 to 8) with whom he has a high degree of interaction (relatives, friends, hunting partners) [35–37]. At any one time, any given individual plays one of two strategies, the ‘old’ technology, or a ‘new’ technology. For each neighbor who plays the same strategy as he does, he gains an interaction-payoff of either 1 (old-old) or *α*_*τ*_ > 1 (new-new). That is, his payoffs in each interaction are given by the coordination game in Table 1. His payoff is then the average of his interaction-payoffs from each neighbor. *α*_*τ*_ represents the within-deme relative fitness benefits of technology *τ* + 1 compared to technology *τ*.

**Table 1:** Payoffs to within-deme interactions, when the deme has current technology level *τ*. *α*_*τ*_ > 1. Entries are interaction-payoffs of an individual whose strategy is given by the row when interacting with an individual whose strategy is given by the column.

	**Old**	**New**
**Old**	1	0
**New**	0	*α*_*τ*_

### Strategy updating

Strategies are updated by single individuals but also by pairs of individuals who can share their intentions. A pair of players can only share intentions if both players in the pair are SI types and they are neighbors on the interaction graph. Each period within a generation, either one individual or one pair of individuals is randomly selected to update their strategy (see Section 2.1 in S1 Supporting Information for precise details). An updating individual or pair of individuals plays a *coalitional better response*, adjusting their strategies so that by doing so they obtain payoffs at least as high as their current payoffs, holding the strategies of all the other individuals fixed [12, 14].

However, when any individual has the opportunity to update his strategy, he will with some small probability make a mistake and switch to a random strategy instead of to his intended strategy [33].

Note that, starting from from a state at which every individual in the population is playing ‘old’, even when a population contains no SI types, the ‘new’ strategy can still be adopted by individuals making mistakes. The neighbors of such mistake-making individuals may then be able to increase their own payoffs by switching to ‘new’. In general, random mistakes, together with better responses, coalitional or individual, allow the spread of the ‘new’ strategy on the interaction graph.

Note that type (SI or N) does not dictate strategy choice, as it does in traditional evolutionary game theory [38, 39]. Neither does type affect any individual’s preferences over profiles of strategies as it does in the literature on evolution of preferences [40]. What type does here is to alter, by enabling or disabling pairwise updating, the set of strategy profiles that can be reached by any given update without mistakes in strategy choice. Note that the SI type only affects behavior in the presence of other SI types and that the behavior manifested by pairs of SI types is mutualistic, in that both participants gain from it (in contrast to altruistic behavior [32, 41–43]).

### Technology adoption

Following strategy updating, payoffs for the current period are realized for all individuals. Following this, if the proportion of the individuals in a deme playing ‘new’ is 90% or higher, we say that the new technology has been adopted, technology *τ* + 1 is now the deme’s current technology, and we reset the strategies of every individual in the deme to ‘old’. The deme has moved one step up the technology ladder.

### Deme extinction

At the end of each generation, with probability *η*, any given deme faces an ‘invader’. The invader is another deme chosen at random. If the invader has a lower technology level than the invaded deme, then the invasion is repelled and nothing further happens. If the invader has a higher technology level, then the invader replaces the invaded deme. The invaded deme becomes extinct and is replaced with a replica (types, current payoffs and tech level) of the invader. If the demes have the same technology, then either outcome occurs with probability one half.

This process can represent the possibility of violent conflict between demes, but can equally be considered to model differing extinction and expansion rates of demes with access to different technology. Low technology demes are more likely to go extinct, whereas high technology demes are more likely to grow and fission into two similar demes. Note that the assumption that unsuccessful demes go completely extinct, though common in papers that feature inter-demic selection (see Table S1 of [43]), can be regarded as unrealistic. The Results section contains a discussion of the robustness of results to a relaxation of this assumption.

### Reproduction

Following the extinction stage, each deme reproduces according to a finite population replicator dynamic with mutation rate *μ*, determining the shares of SI types in the next generation. For simplicity, we assume that reproduction is asexual and haploid. Specifically, the fitness of an individual in a given generation is the sum of his payoffs across all periods within that generation. A parent individual in the current generation is chosen to reproduce with probability proportional to his fitness and his offspring inherits the parent’s type with probability 1 − *μ*. With probability *μ*, a mutation occurs and the offspring’s type differs from that of his parent. *n* offspring are produced independently in this manner and populate the deme for the next generation. Note that as demes comprise finite numbers of individuals, genetic drift will have an effect in demes that contain both SI and N types.

The model was implemented and simulated in the Matlab programming language and run using one of three versions: R2013b, R2014a or R2014b as the project developed. For further details see S1 Supporting Information.

## Results

**Fig 2:**
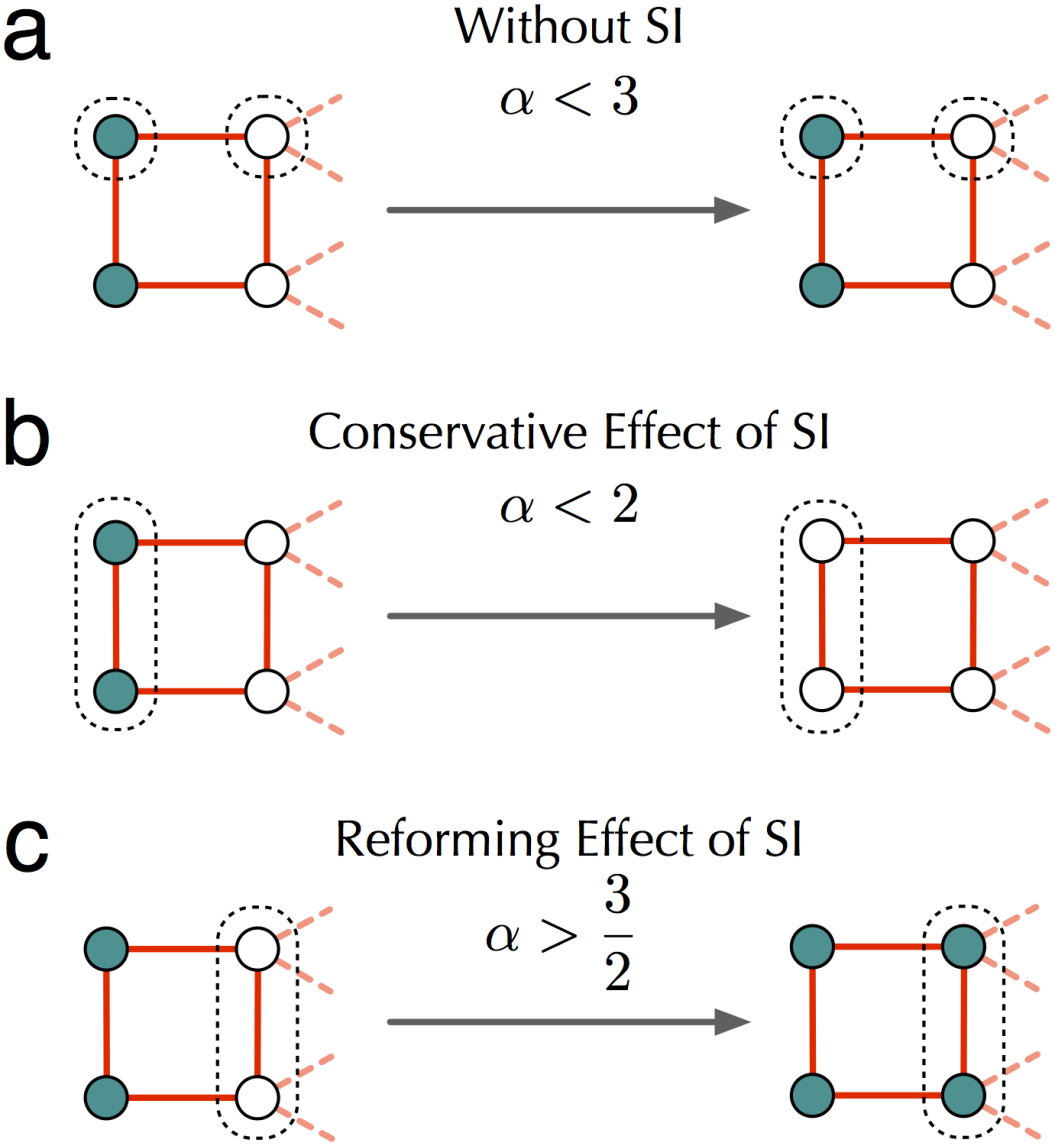
The effect of shared intentionality on technology dynamics. Blue-colored vertices represent individuals playing ‘new’, white vertices represent individuals playing ‘old’. Individuals not shown are assumed to be playing ‘old’. **a:** In the absence of SI, the only better response for any individual is to retain his current strategy. **b:** For low *α*, coalitions of SI type individuals can coordinate payoff improving switches back to ‘old’. **c:** For high *α*, coalitions of SI type individuals can coordinate payoff improving switches to ‘new’ [14]. Note that threshold values of *α* depend on graph structure and that different interaction structures can yield different thresholds [14, 46]. For an example with explicitly calculated thresholds, see Section S2.2.

Relative to demes with low numbers of SI types, demes with high numbers of SI types are slow to adopt new technology when *α*_*τ*_ is low, but fast to adopt new technology when *α*_*τ*_ is high. The former effect arises because, when *α*_*τ*_ is low, SI types playing ‘new’ can coordinate mutually profitable switches back to ‘old’ even when it would not be profitable for any individual acting alone to make such a switch (Fig 2b). Conversely, when *α*_*τ*_ is high, SI types playing ‘old’ can coordinate switches to ‘new’ that would not be profitable for any individual acting alone (Fig 2c). The spread of the ‘new’ strategy across a deme will usually begin with the emergence of small sets of individuals that are playing ‘new’ but are surrounded by individuals who play ‘old’ (Fig 2a). By providing new possibilities for strategic choice, collaboration can destabilize these sets, causing them to contract (Fig 2b) or expand (Fig 2c). Whether it is the former or the latter depends on the relative attractiveness *α*_*τ*_ of the ‘new’ strategy (see Section 2.2 in S1 Supporting Information for worked examples).

Hence, if *α*_*τ*_ remains low (conversely, high) over enough generations, then demes with high numbers of SI types will fall behind (conversely, pull ahead) of demes with low numbers of SI types when it comes to technological advancement. When SI-poor demes lead in technology, they will outperform SI-rich demes, and SI will be selected against in the metapopulation. When SI-rich demes lead in technology, the opposite will occur.

**Fig 3:**
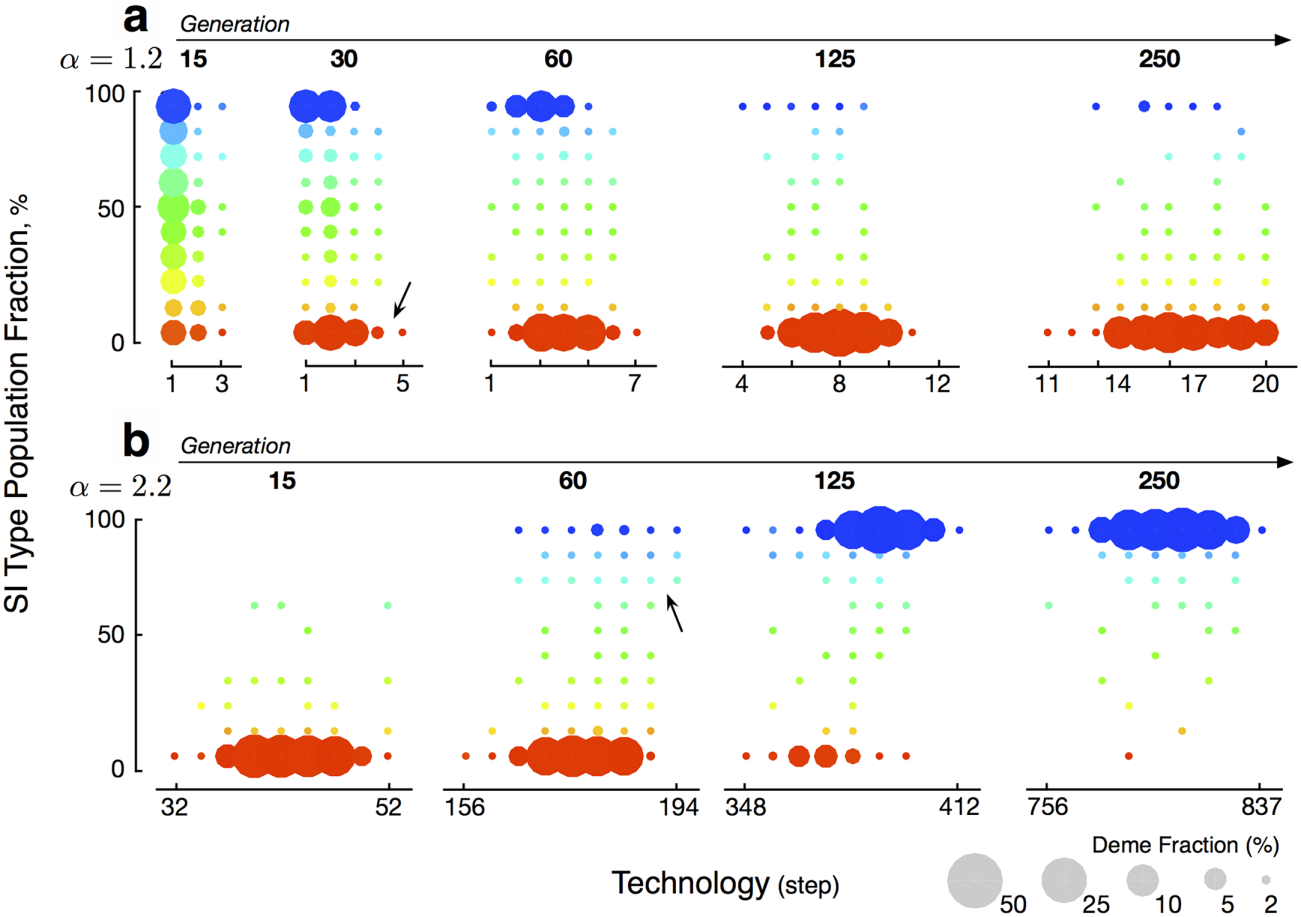
Demes with given fraction of SI type individuals and technology level per generation under benchmark conditions. **a:** *α* = 1.2, starting from a state in which each individual is SI or N type with equal probability, **b:** *α* = 2.2, starting from a state in which no individuals are SI type. Arrows indicate where demes rich in N and SI types respectively gain a technological advantage.

**Fig 4:**
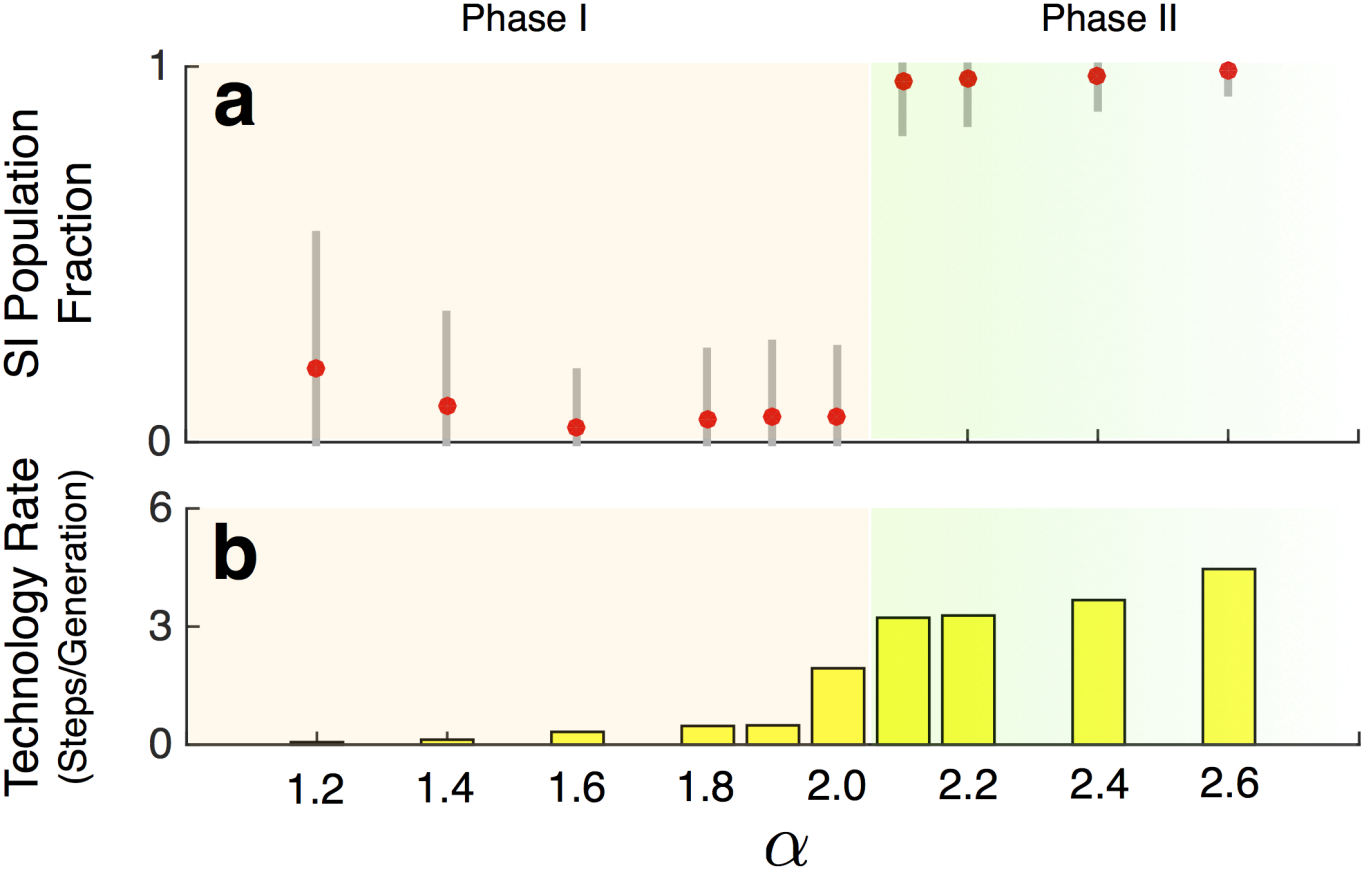
The dramatic switch to a high SI, high technological change, phase for sufficiently high *α*. Starting with each individual equiprobably SI or N type under benchmark conditions, **a:** Mean and standard deviation of fraction of SI type individuals across all 64 demes and 10 replicates during generations 451 to 500, **b:** Average rates of technological change (steps per generation) across all 64 demes and 10 replicates over generations 451 to 500.

When *α*_*τ*_ is low, mutation and genetic drift eventually cause some demes to have low numbers of SI types. These demes gain a technological advantage over other demes, type N becomes dominant and type SI becomes scarce (Figs 3a and 4-Phase I). Conversely, when *α*_*τ*_ is high, mutation, genetic drift, and within deme selection of SI cause some demes to have high numbers of SI types. These demes gain a technological advantage and type SI becomes dominant (Figs 3b and 4-Phase II). These results hold regardless of the initial proportions of SI and N types in the population. Note that the effect of *α* on the share of SI types is not continuous, but rather involves a phase transition. There exists a threshold below which SI is selected against and above which SI is selected for (Fig 4).

The speed of technology adoption increases (discontinuously) in *α* (Fig 4b). This would be the case even without the possibility of the sharing of intentions. However, the switch from a conservative effect of SI (Fig 4-Phase I) to a reforming effect of SI (Fig 4-Phase II) also causes an upwards jump in the rate of technology adoption. The combination of these two effects implies that the emergence and persistence of SI should be accompanied by higher rates of techno-cultural accumulation.

These results are robust to a broad range of parameterizations. Table 2 gives the benchmark parameters that we consider, together with alternative values that we consider in a full factorial robustness check (see Section 5.1 in S1 Supporting Information). Further discussion of, and justification for, the values in Table 2 is given in S1 Supporting Information. Placebo trials demonstrate that inter-demic selection plays the key role in selection against SI (see Figure G in S1 Supporting Information). Conversely, SI can predominate in the metapopulation even without inter-demic selection, although such selection does strengthen the dominance of SI for high *α* treatments.

**Table 2:** Parameter estimates. Benchmark values are in bold.

**Determinant**		**Range**	**Comment/method of estimation**
Number of demes	*m*	**64**	Typical number of languages/dialects in an AIATSIS linguistic zone [S1]
Effective deme size (one-third of census size)	*n*	**32**	Per previous estimates [17, 37]
Average number of neighbors per individual	*d*	4, **6**, 8	Degree of scale-free graph (social network) [S1]
Within-deme fitness benefits of new technology	*α*	1.2–4.0	Depends on technology/norm under consideration
Periods per generation	*T*	**2000**	2 updates per week over 20 years
Maximum coalition size for strategy updating	*k*	**2**, 3, 4	Pairwise updating (benchmark) and small coalitions [46] [S1]
Mistake rate in strategy updating	*ɛ*	0.025, **0.05**, 0.10	One mistake every 10–40 updates (benchmark 20)
Mutation rate from SI to N and vice versa	*μ*	**0.001**	For simulation purposes. For lower rates adjust timescales accordingly
Per generation ‘conflict’ probability	*η*	0.05, **0.10**, 0.15	Similar to previous estimates [17] [S1]

We further test robustness of results to two other variations. Firstly, we allow migration of individuals between demes (see S1 Supporting Information, Section 5.4). Secondly, we amend the Deme Extinction stage so that the population of an invaded low technology deme is only partially replaced by replica members of an invading high technology deme (see S1 Supporting Information, Section 5.5). Selection both for and against SI is robust to some variation along both of these dimensions. In line with the placebo trials, selection against SI disappears if inter-demic selection is sufficiently weakened (by either migration or partial replacement), but selection for SI is consistently robust.

## Discussion

As might be expected, large gains from technological adaptation facilitate the evolution of SI. However, when benefits from new technology are low, collaboration works against a community by slowing its technological advance, even when all members of the community have perfectly aligned interests. Previous literature has discussed how interaction structure can have important implications for cumulative culture [37, 44, 45]. Our model provides a novel mechanism for this: the social structure within demes combines with the presence or absence of shared intentions and the exogenous technological opportunities of the epoch (*α*) to give varying rates of techno-cultural accumulation. Furthermore, although this study considers the plausible case of scale free social networks [36], there exist a large range of social structures, both regular and random, for which the ‘conservative’ and ‘reforming’ effects of Fig 2 are observed [14, 46].

Thus our model gives a mechanism by which inter-species (e.g. chimp vs. human) differences in benefits from new technologies could lead to diversity in the ability to share intentions. Such differences in the gains from technological advance could arise from physical differences between species, or from differences in environmental variability [26, 27]. Our results indicate that an extended period of environmental change leading to elevated within-deme fitness benefits from innovation would have sufficed for SI to become widespread.

It has been shown that even when we restrict our attention to coordination games, the evolution of jointly intentional behavior is not guaranteed. For other games, such as prisoner’s dilemmas, we conjecture that the conditions for its emergence will be stricter. Further careful consideration should lead to other such hypotheses as well as to interpretations of existing data. The authors suspect that sometimes the theory of collaborative strategic choice will complement theories of altruism, but sometimes it will compete.

## Supporting Information

S1 Supporting Information Containing detailed discussion, methods, and robustness checks is attached to this submission. (PDF)

## References

[1] Call J. Contrasting the social cognition of humans and nonhuman apes: The shared intentionality hypothesis. Topics in Cognitive Science. 2009;1(2):368–379. 25164939 10.1111/j.1756-8765.2009.01025.x

[2] Vygotsky LS. Mind in society: The development of higher psychological processes. Harvard University Press; 1980.

[3] Tomasello M. A natural history of human thinking. Harvard University Press; 2014.

[4] Moll H, Tomasello M. Cooperation and human cognition: the Vygotskian intelligence hypothesis. Philosophical Transactions of the Royal Society B: Biological Sciences. 2007;362(1480):639–648. 10.1098/rstb.2006.2000 PMC2346522 17296598

[5] Tomasello M, Herrmann E. Ape and Human Cognition What’s the Difference? Current Directions in Psychological Science. 2010;19(1):3–8. 10.1177/0963721409359300

[6] Tomasello M, Carpenter M. Shared intentionality. Developmental science. 2007;10(1):121–125. 10.1111/j.1467-7687.2007.00573.x 17181709

[7] Tuomela R, Miller K. We-intentions. Philosophical Studies. 1988;53(3):367–389. 10.1007/BF00353512

[8] Searle J. Collective intentions and actions In: Cohen PR, Morgan J, Pollack M, editors. Intentions in communication. MIT Press; 1990 p. 401–15.

[9] Bratman ME. Shared cooperative activity. The Philosophical Review. 1992;101(2):327–341. 10.2307/2185537

[10] Gilbert M. Walking together: A paradigmatic social phenomenon. Midwest Studies in Philosophy. 1990;15(1):1–14. 10.1111/j.1475-4975.1990.tb00202.x

[11] Newton J. Coalitional stochastic stability. Games and Economic Behavior. 2012;75(2):842–54. 10.1016/j.geb.2012.02.014

[12] Newton J. Recontracting and stochastic stability in cooperative games. Journal of Economic Theory. 2012 1;147(1):364–81. 10.1016/j.jet.2011.11.007

[13] Sawa R. Coalitional Stochastic Stability in Games, Networks and Markets. Games and Economic Behavior. 2014;88:90–111. 10.1016/j.geb.2014.07.005

[14] Newton J, Angus SD. Coalitions, tipping points and the speed of evolution. Journal of Economic Theory. 2015;157(0):172–187. 10.1016/j.jet.2015.01.003

[15] Tomasello M, Melis AP, Tennie C, Wyman E, Herrmann E. Two key steps in the evolution of human cooperation. Current Anthropology. 2012;53(6):673–692. 10.1086/668207

[16] Sterelny K. Cooperation, Culture, and Conflict. The British Journal for the Philosophy of Science. 2014;p. axu024.

[17] Bowles S. Group competition, reproductive leveling, and the evolution of human altruism. Science. 2006;314(5805):1569–1572. 10.1126/science.1134829 17158320

[18] Choi JK, Bowles S. The coevolution of parochial altruism and war. Science. 2007;318(5850):636–640. 10.1126/science.1144237 17962562

[19] Schelling TC. The strategy of conflict. Reprint 1980, Harvard university press; 1960.

[20] Ellison G. Learning, Local Interaction, and Coordination. Econometrica. 1993 9;61(5):1047–71. 10.2307/2951493

[21] Young HP. Individual strategy and social structure. Princeton University Press; 1998.

[22] Ellison G. Basins of Attraction, Long-Run Stochastic Stability, and the Speed of Step-by-Step Evolution. Review of Economic Studies. 2000 1;67(1):17–45. 10.1111/1467-937X.00119

[23] Montanari A, Saberi A. The spread of innovations in social networks. Proceedings of the National Academy of Sciences. 2010 11;107(47):20196–20201. 10.1073/pnas.1004098107 PMC2996710 21076030

[24] Young HP. The dynamics of social innovation. Proceedings of the National Academy of Sciences. 2011;108 Suppl 4:21285–91. 10.1073/pnas.1100973108 PMC3271568 22198762

[25] Kreindler GE, Young HP. Rapid innovation diffusion in social networks. Proceedings of the National Academy of Sciences. 2014;111(Supplement 3):10881–10888. 10.1073/pnas.1400842111 PMC4113916 25024191

[26] Potts R. Evolution and climate variability. Science. 1996;273(5277):922–923. 10.1126/science.273.5277.922

[27] Potts R. Hominin evolution in settings of strong environmental variability. Quaternary Science Reviews. 2013;73:1–13. 10.1016/j.quascirev.2013.04.003

[28] Ambrose SH. Paleolithic technology and human evolution. Science. 2001;291(5509):1748–1753. 10.1126/science.1059487 11249821

[29] Whiten A, Van Schaik CP. The evolution of animal ‘cultures’ and social intelligence. Philosophical Transactions of the Royal Society B: Biological Sciences. 2007;362(1480):603–620. 10.1098/rstb.2006.1998 PMC2346520 17255007

[30] van Schaik CP, Burkart JM. Social learning and evolution: the cultural intelligence hypothesis. Philosophical Transactions of the Royal Society of London B: Biological Sciences. 2011;366(1567):1008–1016. 10.1098/rstb.2010.0304 21357223 PMC3049085

[31] Bacharach M. Beyond individual choice: teams and frames in game theory. Princeton University Press; 2006.

[32] Szathmary E, MaynardÃ‚ Smith J. The major transitions in evolution. Oxford University Press; 1995.

[33] Young HP. The Evolution of Conventions. Econometrica. 1993 1;61(1):57–84. 10.2307/2951778

[34] Gavrilets S. Collective action and the collaborative brain. Journal of The Royal Society Interface. 2014;12(102). 10.1098/rsif.2014.1067 PMC4277098 25551149

[35] Ohtsuki H, Hauert C, Lieberman E, Nowak MA. A simple rule for the evolution of cooperation on graphs and social networks. Nature. 2006;441(7092):502–505. 10.1038/nature04605 16724065 PMC2430087

[36] Apicella CL, Marlowe FW, Fowler JH, Christakis NA. Social networks and cooperation in hunter-gatherers. Nature. 2012 1;481(7382):497–501. 10.1038/nature10736 22281599 PMC3340565

[37] Hill KR, Walker RS, Božičević M, Eder J, Headland T, Hewlett B, et al Co-Residence Patterns in Hunter-Gatherer Societies Show Unique Human Social Structure. Science. 2011 3;331(6022):1286–1289. 10.1126/science.1199071 21393537

[38] Weibull JW. Evolutionary game theory. MIT press; 1997.

[39] Dugatkin LA, Reeve HK. Game theory and animal behavior. Oxford University Press; 1998.

[40] Güth W, Kliemt H. The indirect evolutionary approach: Bridging the gap between rationality and adaptation. Rationality and Society. 1998;10(3):377–399. 10.1177/104346398010003005

[41] Alvard MS, Nolin DA. Rousseau’s Whale Hunt? Current Anthropology. 2002;43(4):533–559. 10.1086/341653

[42] Smith EA. Human Cooperation: Perspectives from Behavioral Ecology In: Hammerstein P, editor. Genetic and cultural evolution of cooperation. MIT Press; 2003 p. 401–427.

[43] Rusch H. The evolutionary interplay of intergroup conflict and altruism in humans: a review of parochial altruism theory and prospects for its extension. Proceedings of the Royal Society B: Biological Sciences. 2014;281(1794):20141539 10.1098/rspb.2014.1539 25253457 PMC4211448

[44] Boyd R, Richerson PJ. Group Beneficial Norms Can Spread Rapidly in a Structured Population. Journal of Theoretical Biology. 2002;215(3):287–296. 10.1006/jtbi.2001.2515 12054837

[45] Hill KR, Wood BM, Baggio J, Hurtado AM, Boyd RT. Hunter-gatherer inter-band interaction rates: Implications for cumulative culture. PloS One. 2014;9(7):e102806 10.1371/journal.pone.0102806 25047714 PMC4105570

[46] Newton J, Angus S. Coalitions, tipping points and the speed of evolution. University of Sydney Economics Working Paper Series. 2013;2013–02.

