# Supplementary material for: Emergence of Shared Intentionality Is Coupled to the Advance of Cumulative Culture
Source: PLoS Comput Biol. 2015 Oct 30;11(10):e1004587. doi: 10.1371/journal.pcbi.1004587 (PMC4627749; doi:10.1371/journal.pcbi.1004587)
Supplement: S1 Supporting Information — (PDF) [file pcbi.1004587.s001.pdf]

Emergence of shared intentionality is coupled to the advance of cumulative culture

Angus & Newton, PLoS - Computational Biology, 2015

S1 Supporting Information

Contents

1 Shared intentions: discussion **1**
- 1.1 Philosophy . . . 2
- 1.2 Psychology . . . 2
- 1.3 Game theory . . . 3
- 1.4 Mutualism vs. altruism . . . 4

2 Multi-level selection **4**
- 2.1 Strategy updating . . . 4
- 2.2 Deriving thresholds for example networks . . . 5
- 2.3 Reproduction and selection within demes . . . 6
- 2.4 Deme extinction and survival . . . 7
- 2.5 Deme extinction: examples . . . 7

3 Choice of benchmark parameters **9**

4 Social networks **11**
- 4.1 Network type & Characteristics . . . 11
- 4.2 Coalition formation . . . 13

5 Additional Experiments: Robustness, the long run & placebo trials **13**
- 5.1 Robustness . . . 13
- 5.2 The long run . . . 16
- 5.3 Placebo Trials . . . 17
- 5.4 Migration . . . 19
- 5.5 'Brutality' in conflict and replacement . . . 19

6 Numerical simulation **24**

1 Shared intentions: discussion

Here we relate the current study to work on shared intentions and collaborative behaviour that has been published in the fields of philosophy, developmental psychology and game theory. Philosophy is foundational to considering joint action and the sharing of intentions. The evidence from psychology is motivational, in that it stresses the importance of collaboration to human development, even going so far as to suggest that it is the ability to share intentions and collaborate which separates humans from the other great apes. Game theory is the methodological tool that we choose to use and adapt to model the implications of sharing an intention and behaving collaboratively. Finally, we consider the difference between altruistic actions and the mutualism of collaboratively sharing intentions.

1.1 Philosophy

It has been argued in the philosophical literature that the intentions behind collective acts can be distinct from an aggregation of individual intentions (Bratman, 1992; Searle, 1990). This is *shared/collective intentionality*, the idea that "we will do X" is distinct from "I will do X [because she is also doing X]". Collective action can occur through either mode of reasoning. Consider the following two player game. It is a coordination game in which the two players (me and you) only achieve payoffs if they coordinate on the same action. Given coordination on the same action, the players would rather that this action be action A.

	A	B
A	2	0
B	0	1

When it comes to describing *actions* in such a game, an expression such as (W1) "We are performing action B" can be considered equivalent to an expression such as (I1) "I am performing action B and you are performing action B". However, when it comes to describing *intentions*, there can be no such equivalence. Consider the expressions (W2) "We intend to do B" and (I2) " I intend to do B and I think that you intend to do B".[1] It can be argued that, given that the players know the payoffs of the game, that (W2) does not make sense because the intended action profile is Pareto inefficient: both players could do better if they instead intended to do A. Moreover, such an alternative intention would be *self enforcing* in that given a shared intention to do A, neither of the players need worry about a subsequent deviation by a cheating partner. However, such an argument does not apply to (I2). If I truly believe that you intend to do B, then it is optimal for me to do B (and vice versa), regardless of the Pareto inefficiency of the action profile (B,B). To phrase this another way, in the context of optimization, individual intentions give rise to individual optimality constraints, whereas shared intentions give rise to joint optimality constraints. In the latter case, the object which is chosen optimally is a vector of the actions of multiple players.[2]

There has been debate amongst philosophers as to whether shared intentions can always be reduced to individual intentions plus beliefs about the intentions of others (Bratman, 1992; Butterfill, 2012; Gilbert, 1990; Gold and Sugden, 2007; Searle, 1990; Tuomela and Miller, 1988; Velleman, 1997). The current paper shows that the behaviour implied by the sharing of intentions, that is the joint optimization of action choice, can evolve in the absence of hierarchical beliefs (I think that you think that I think...) and other complex modes of reasoning.

1.2 Psychology

The current study is further motivated by recent work in developmental psychology. Experiments with children suggest that the collaborative urge in humans is a primal one, which develops early in infancy, prior to much of our aptitude for rational inference, and certainly prior to our ability to articulate complex hierarchical beliefs such as are required by traditional game theory (See Tomasello and Rakoczy, 2003 for a summary, as well as Tomasello et al., 2005 and the critical responses contained therein). Moreover, this collaborative urge is considerably weaker in non-human great apes (Tomasello and Carpenter, 2007;

[1]Searle (1980) distinguishes between *prior intentions*, which are held ahead of the intended action, and *intentions in action*, which are held contemporaneously with the intended action being carried out. Considering this latter type of intention, in the notation of the cited paper, the *presentational content* of the intention which forms part of (I2) is

(B as a result of this intention in action).

Similarly, we can write the presentational content of the intention in (W2) as

((B,B) as a result of this intention in action).

Note that the intentions are similarly causal with respect to their intended actions. The difference is that the latter of the two intentions, the shared intention, has a vector of actions by multiple players as its intended outcome.

[2]Gold and Sugden (2007) go further, arguing that it is the process by which an intention is formed that makes it collective (shared) or not. They suggest modes of reasoning by which collective intentions may be formed. These modes of reasoning all involve the satisfaction of joint optimality constraints.

Tomasello and Herrmann, 2010; Wobber et al., 2014). To summarize in the words of Tomasello (2014):

> ...humans are able to coordinate with others, in a way that other primates seemingly are not, to form a "we" that acts as a kind of plural agent to create everything from a collaborative hunting party to a cultural institution. (1)

This accumulated evidence has provided support for the hypothesis that the human ability to collaborate led to the development of our unique cognitive skill set, rather than causality running only in the opposite direction. In short, human collaborative activity provided a niche in which sophisticated modes of reasoning could evolve. This is known as as the shared intentionality hypothesis (Call, 2009) or the Vygotskian intelligence hypothesis (Moll and Tomasello, 2007; Tomasello, 2014; Vygotsky, 1980).

The principal philosophical work to which Tomasello and his coauthors make reference when discussing the sharing of intentions is the work of Bratman (1992). However, although Bratman does not argue that shared intentions can be wholly described by individual intentions plus beliefs, he does make use of the notion of common knowledge (I know that you know that I know...ad infinitum) in his discussion of shared cooperative activity. Of course, common knowledge requires hierarchical thought and thus a considerable degree of sophistication in reasoning. This raises a potential problem of circularity: if collaboration requires sophisticated reasoning, how could collaboration have arisen prior to such reasoning? Our work addresses this problem with a very clear answer. Consistent with the philosophical analysis discussed in Section 1.1 and with the psychological evidence summarized in statement (1), we model the sharing of intentions as collective agency: individuals who can share intentions will sometimes come together and adjust their actions to their mutual benefit. That is, they engage in joint strategic choice.

(i) We show that joint goal oriented behaviour, as implied by shared intentions, could evolve prior to sophisticated reasoning (our agents are myopic optimizers). This is plausible: developmental studies of children indicate that they can undertake intentional action at earlier ages than they can understand beliefs (Baron-Cohen, 1994; Call and Tomasello, 1999; Carpenter et al., 1998a,b; Wellman and Bartsch, 1994; Wellman et al., 2001).

(ii) Moreover, we show conditions under which such joint behaviour would not evolve. That is, the ability to collaborate is not always an unambiguous good. Sometimes the short term benefits to collaboration can work to the long term detriment of a society. In a model of multi-level selection this can work against the evolution of a collaborative disposition.

The above points show that the ability to collaborate and share intentions could be selected for or against differently, depending on geographical location, ecological conditions, climate variability and species. The last of these is particularly important, as any story that explains how humans could become the collaborative species we are today, should explain how it could be that this is less so in other great apes.

1.3 Game theory

There exists a large literature in cooperative game theory on the behaviour of coalitions. For a survey the reader is referred to Peleg and Sudholter (2003). There is a smaller but established literature at the intersection of noncooperative and cooperative game theory (See, for example Ambrus, 2009; Aumann, 1959; Bacharach, 2006; Bernheim et al., 1987; Konishi and Ray, 2003; Luo and Yang, 2009). There is also a large and established literature in evolutionary game theory that seeks to explain altruism ('cooperation'), where altruism manifests itself through the actions that players choose (See Eshel and Cavalli-Sforza, 1982; Nowak, 2006; Wilson and Dugatkin, 1997, and references contained therein). The sharing of intentions and collaboration is, however, different from altruism. Rather than manifest itself through the identity of chosen actions, as altruism does, shared intentions manifest themselves through *how* actions are chosen. Specifically, when actions are chosen, collaborating players optimize over joint strategy choice. This does not imply any kind of concern for the well-being of others, merely an efficient (in the short term) way of attaining higher payoffs in conjuction with other players. Work on the incorporation of such collaborative, coalitional behaviour into evolutionary dynamics forms a relatively new and rapidly growing literature (Newton, 2012a,b; Sawa, 2014), although considerable work has previously been done in the context of matching, which in effect concerns coalitions of size two (Diamantoudi et al., 2004; Jackson and Watts,

2002; Klaus et al., 2010; Newton and Sawa, 2015; Roth and Vande Vate, 1990). The result, used in the current study, that strategic updating by coalitions can both slow (the conservative effect) and hasten (the reforming effect) the convergence of a population to an efficient 'new' action, was first analyzed in Newton and Angus (2013, 2015). The cited work contains an in depth analysis of such effects, showing that they are supported on a wide variety of networks beyond the scale free networks that are used in the current study.

1.4 Mutualism vs. altruism

The current study concerns the evolution of the sharing of intentions and collaboration by individuals *to their mutual benefit*. That is, the sharing of intentions is a mutualistic activity and not an altruistic one, although an individual who shares his intentions could in theory have altruistic motives. Models of multi-level selection are by now standard in the literature on the evolution of altruism (See Bowles, 2006; Traulsen and Nowak, 2006). A recent survey and critique of this literature can be found in Rusch (2014). We use multi-level selection to model the evolution, and non-evolution, of the mutualistic behaviour that is collaborative action choice in a game. To model this without confounding issues of altruism we use a simple coordination game as the underlying game.

	**Old**	**New**
**Old**	1	0
**New**	0	α_τ

Table A: Payoffs to within-deme interactions, when the deme has current technology level τ. $\alpha_\tau > 1$. Entries are interaction-payoffs of an individual whose strategy is given by the row when interacting with an individual whose strategy is given by the column.

The altruism literature focuses on explaining efficient coordination in prisoner's dilemma type situations. The question of whether efficient collaboration would always evolve in situations in which individuals' interests are perfectly aligned has, to the best of our knowledge, not been addressed. West et al. (2007) distinguish four types of behaviour: selfishness - actions that benefit oneself but harm others $(+,-)$, mutualism $(+,+)$, altruism $(-,+)$ and spite $(-,-)$. Rusch (2014) notes that selfishness and mutualism are often taken for granted, with research focusing on the evolution of altruism and spite. The current study indicates that this neglect of the evolution of mutualism should be reconsidered, and that pairwise and small-group mutualistic interaction (coalitional updating) can sometimes work to the detriment of the welfare of the larger society (the deme).

It is thought that many instances of collaboration amongst hunter gatherers are mutualistic. Examples include hunting for whales and other large animals (Alvard, 2001; Alvard and Nolin, 2002) and fishing expeditions (Sosis et al., 1998). See Smith (2003) for a survey. It has been subsequently noted by Bird et al. (2012) that group hunting may not always be mutualistic for all members of a hunting party as, for example, sharing norms may disproportionately reduce the take of the best hunters. However, despite the usefulness of modern anthropological evidence, our model does not concern modern hunter-gatherers, and the authors find it hard to conceive of a species developing sophisticated egalitarian cultural institutions prior to the ability to collaborate in simple mutualistic situations.

2 Multi-level selection

2.1 Strategy updating

Strategies are updated by single individuals but also by pairs of individuals who can share their intentions. A pair of players can only share intentions if both players in the pair are SI types and they are neighbours on the interaction graph. Each period within a generation, either one individual or one pair of individuals is randomly selected to update their strategy.

The algorithm by which the set of updating individuals is selected is as follows. Firstly, a single individual is selected uniformly at random from all of the individuals in the deme. Let this individual be labeled i. If individual i is an N type, then he will update his strategy on his own. We return the set of

updating individuals $\{i\}$. If individual i is an SI type, then uniformly at random choose $\bar{k} \in \{1, 2\}$. If $\bar{k} = 1$, then return the set of updating individuals $\{i\}$. If $\bar{k} = 2$, then uniformly at random choose a set of players from the set of all feasible coalitions and return this set as the set of updating individuals. For example, if i has three neighbors who are SI types, j_1, j_2 and j_3, then there are four feasible coalitions: $\{i\}, \{i, j_1\}, \{i, j_2\}, \{i, j_3\}$, any of which may be returned as the set of updating individuals.

Note that due to combinatoric considerations, when there are many SI types in a deme, the number of feasible coalitions of size $\bar{k}$ grows rapidly in $\bar{k}$. Therefore, it is to avoid a strong bias in favour of larger coalitions that we select the maximum coalition size $\bar{k}$ before randomizing over possible coalitions.

When a single individual (i.e. not a pair) is selected, he plays a *better response* to the strategies of the other individuals. That is, holding the strategies of all other individuals fixed, he chooses some strategy ('old' or 'new') that gives him a payoff at least as high as his current payoff. If both strategies give a payoff at least as high as the current payoff, then he chooses uniformly at random.

When a pair of neighbouring SI types is chosen to update their strategies, they can share intentions and collaborate in choosing identical strategies (both choose 'old' or both choose 'new'), but will only do so if by doing so they obtain payoffs at least as high as their current payoffs, holding the strategies of all other individuals fixed. If no such opportunity for successful collaboration exists, both individuals remain playing the same strategy as before. If there are multiple opportunities for successful collaboration; that is, both individuals switching to 'old' and both individuals switching to 'new' would weakly increase the payoffs of both individuals in the pair, then either of these possibilities occurs with probability one half. In summary, the pair plays a *coalitional better response* (Newton, 2012b; Newton and Angus, 2015).

In our comparison treatments we sometimes include the possibility of updating coalitions of players that contain more than two players; that is, $k > 2$. For these treatments, every member of a coalition chosen to update must be an SI type. Furthermore, coalitions chosen to update must be connected in the following way: the subgraph induced by restricting the interaction graph to the vertices corresponding to the individuals in the given coalition must be connected. The interpretation of this is that individuals who share their intentions as part of a coalition must have some form of communication and interaction in order for them to do so. Note that for $k = 2$, this description of feasible coalitions reduces to our previous definition of feasible coalitions as neighbouring pairs on the interaction graph. The process of selecting an updating coalition is as above, but with $\bar{k}$ being selected uniformly at random from $\{1, \ldots, k\}$ instead of from $\{1, 2\}$. Coalitions of size $k > 2$ play coalitional better responses in a similar manner to coalitions of size $k = 2$ as described above (replacing 'both individuals switching' with 'all individuals in the coalition switching').

Finally, when any player has the opportunity to update his strategy, we allow him to make a mistake: independently with small probability ε any updating individual switches to a random strategy instead of to their intended strategy (Young, 1993).

2.2 Deriving thresholds for example networks

Here we illustrate threshold effects for conservative and reforming effects of SI through a discussion of two example networks, the network of overlapping triangles of Figure A(a) and the square lattice with von Neumann neighborhood of Figure A(b). For the purpose of this example we shall only consider $\alpha < 3$. Consider the situation illustrated in Figure A(a) in which all individuals are playing strategy 'old' except for the individual labeled **a**, the individual labeled **b**, and their mutual neighbor. Note that such a strategy profile can be reached irrespective of whether any of the individuals in the population are SI types. For example, starting from a profile at which every player plays 'old', it might be the case that individual **a** has an updating opportunity, makes a mistake (with probability ε), and switches to 'new' when his best response is to remain playing 'old'. Individual **b** could make a similar mistake, switching to 'new', following which the best response of the mutual neighbor of **a** and **b**, when called upon to update, would be to switch to 'new'.

Consider the individual labeled **a** in Figure A(a). Let o_a be the number of his neighbors who play 'old', and n_a be the number of his neighbors who play 'new'. If individual **a** is selected to update his strategy as an individual (whether he is an N or an SI type), he will gain from switching from 'new' to 'old' whenever $\alpha n_a < o_a$. Now, at the strategy profile depicted in Figure A(a), $o_a = 2$ and $n_a = 2$, so this condition becomes $\alpha < 1$, which is impossible. Hence **a** will remain playing 'new'. Now, consider the case that

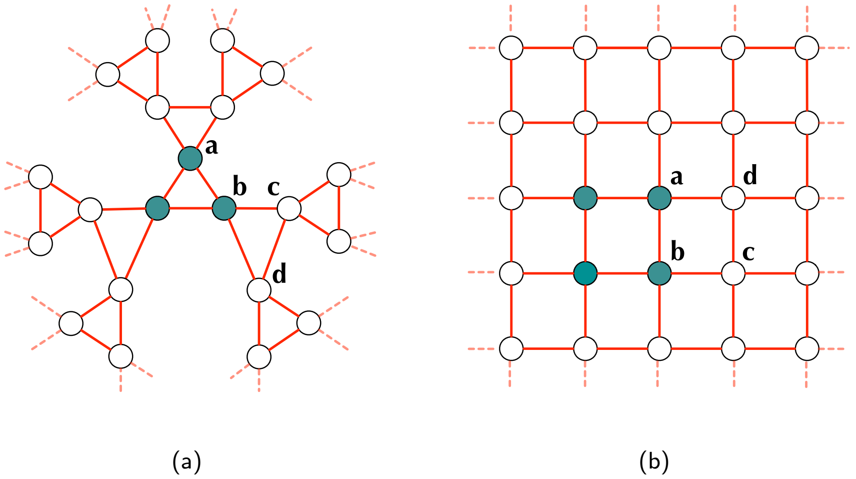

Figure A: **Example networks.** Individuals playing 'new' indicated by filled circles.

neighboring individuals **a** and **b** are both SI types and are selected to update strategies as a pair. They will both gain from switching from 'new' to 'old' if $\alpha n_a < o_a + 1$ and $\alpha n_b < o_b + 1$. As $o_a = o_b = n_a = n_b = 2$, these conditions both become $\alpha < 3/2$. So, from the strategy profile depicted in Figure **A**(a), if $\alpha < 3/2$, the presence of SI types can cause a reduction in the number of individuals choosing 'new'. The cluster of individuals playing the new technology can be eradicated by coalitional better responses.

Now, consider the individual labeled **c** in Figure **A**(a). Let o_c be the number of his neighbors who play 'old', and n_c be the number of his neighbors who play 'new'. If individual **c** is selected to update his strategy as an individual (whether he is an N or an SI type), he will gain from switching from 'old' to 'new' whenever $\alpha n_c > o_c$. Now, at the strategy profile depicted in Figure **A**(a), $o_c = 3$ and $n_c = 1$, so this condition becomes $\alpha > 3$. Hence **c** will remain playing 'old' unless α is very high. Now, consider the case that neighboring individuals **c** and **d** are both SI types and are selected to update strategies as a pair. They will both gain from switching from 'old' to 'new' if $\alpha(n_c + 1) > o_c$ and $\alpha(n_d + 1) > o_d$. As $o_c = o_d = 3$ and $n_c = n_d = 1$, these conditions both become $\alpha > 3/2$. So, from the strategy profile depicted in Figure **A**(a), if $\alpha > 3/2$, the presence of SI types can cause additional pairs of individuals adjacent to the set of individuals choosing 'new' to switch to 'new'. The set of individuals playing the new technology can be expanded by coalitional better responses.

Hence, we see that for the network of overlapping triangles, an important threshold value is $\alpha = 3/2$. For values of α below this threshold, SI slows the adoption of new technologies. For values of α above this threshold, SI speeds the adoption of new technologies. The argument above can be repeated almost word for word for the square lattice with von Neumann neighborhood with strategy profile and labeling of individuals given by Figure **A**(b). For precise, analytic results for vanishing mistake rates ($\varepsilon \to 0$) we refer the reader to Newton and Angus (2013, 2015). The cited work also illustrates the difficulties in determining analytically the values of thresholds for general, more realistic social networks such as the ones used in the current study.

2.3 Reproduction and selection within demes

Each period within a generation, the payoffs of each individual in a deme are determined by the payoffs in the game in Table **A**. For example, consider a period in which a deme is at technology level τ. Consider an individual within that deme who is playing 'new' and has three neighbours playing 'new' and two neighbours playing 'old'. The payoff of this individual will be $3\alpha_\tau/5$, his average payoff across all of his neighbours. The fitness of the individual in a given generation is the sum of these payoffs across every period in the generation. A fitness vector is constructed in which the fitness of individual i, $1 \leq i \leq n$ within the deme is the ith entry in the vector. The vector is normalized so that it is a unit vector. Then the value in the ith element of the vector gives the probability with which any given child born into the next generation

will be the offspring of individual i. To illustrate, assume we have a fitness vector $(p_1, p_2, \ldots, p_n)$. Then, any given individual born into the next generation will, independently, be the offspring of individual i with probability p_i. With probability $1 - \mu$, the offspring of individual i will have the same type, SI or N, as individual i. However, with probability μ any given offspring will undergo a mutation and be the opposite type to her parent. In this manner, all n positions in the deme for the subsequent generation are filled.

Note that the replication here is nothing more than a discretized version of the replicator dynamic. Naturally, in the finite setting, there are some differences to the continuous population replicator dynamic. The clearest difference is that even without considering selection or mutation, when starting from any mixed population, the randomness in reproduction, genetic drift, can eliminate either of the types from the population. Genetic drift assists in creating variation in the proportions of types within demes, so that higher level selection between demes can then take place.

α_τ should be understood as the within-deme relative fitness benefit of using technology $\tau + 1$ rather than technology τ. Payoffs and fitness within demes depend directly on α. Unlike the model of Bowles (2006), we do not assume the presence of an egalitarian food sharing norm. Such a norm, if it did exist, would enter our model through weaker within-deme selective pressure. Our simulations show that selective pressure within demes acts in favour of SI (Figure **G**). This effect would be weaker if egalitarian norms existed.

2.4 Deme extinction and survival

Each generation, any given deme, with probability η, faces an invader who is one of the other demes drawn at random. If the incumbent deme has higher technology than the invader, then nothing changes. If the invader has higher technology than the incumbent, then the incumbent deme is eliminated and its place is taken by a replica of the invading deme (same number of SI and N types, same technology level, same payoffs). If the incumbent and the invader have the same technology level, then the incumbent is replaced with probability one half.

Note that α affects inter-demic contests only indirectly through its effect on technology adoption. It is possible to set up the model differently, so that rather than old and new technologies giving payoffs of 1 and α_τ respectively, they give payoffs of α_τ and $\alpha_{\tau+1}$, with higher values of τ associated with higher values of α_τ. Group fitness can then be made to depend on cumulative fitnesses of individuals in the deme. Test simulations indicated that this approach gives similar results to our chosen approach, but is significantly more computationally demanding.

We examine $\eta = 0.05, \underline{0.10}, 0.20$, with the middle value being our benchmark value. This value, $\eta = 0.10$, corresponds to a deme extinction rate of approximately $\eta/2 = 0.05$ per generation. This is less than the benchmark rate of 0.075 used in Bowles (2006). That is, the benchmark rates of 'conflict' used in the current study are lower than those used in the most comparable existing study. Furthermore, our results are robust to lower and higher conflict probabilities. We refer the interested reader to the Supporting Online Material to Bowles (2006) for arguments and references in support of such rates of conflict. Key citations therein include Frayer and Martin (2014); Hill and Hurtado (1996); Keeley (1996); Kelly (2000).

Importantly, unlike Bowles (2006); Choi and Bowles (2007), where a higher prevalence of altruistic types is assumed to lead to greater success in conflict, the type of individuals (SI or N) in the current study has no direct effect on probabilities of deme extinction or duplication. We do not, in the words of Sterelny (2014), 'lump the civic and the military virtues'. This means that, although the deme extinction and replacement events of the current study can be understood as the outcome of conflict, they can also be understood as representing the outcome of bloodless competition over land or resources in which higher technology gives an advantage, or simply higher survival (conversely, extinction) rates for demes with higher (conversely, lower) technology.

2.5 Deme extinction: examples

In Figure **B** and **C** we provide example visualisations of inter-deme contests resulting in deme extinction and the type evolution of the population at the conclusion of each of three generations. (Note, other contests, not shown, may have occured resulting in the survival of the incumbent.)

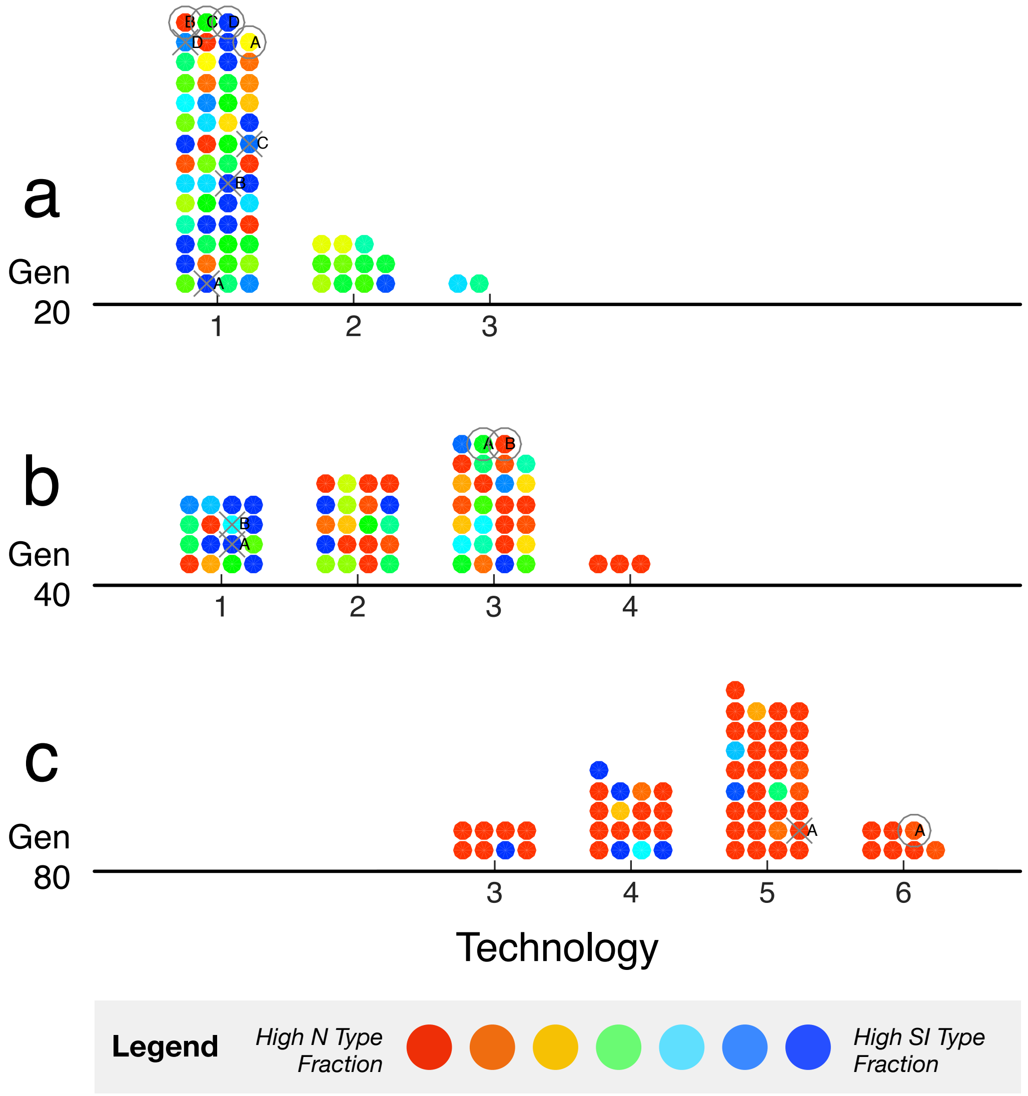

Figure B: **Example deme extinction and type evolution visualisation at benchmark parameter settings and $\alpha = 1.2$ (single replicate).** Each disc represents a single deme, 64 demes in all, and is coloured according to the fraction of SI (or N) types within it (see Legend for scheme). Discs are positioned according to the technology level obtained by the at the conlusion of the generation. There is no significance to the height-positioning of demes. Material deme-deme conflict events are labelled $(A, B, \ldots)$ and indicate the invaded deme $(\times)$ and its replacement (enclosed). The outcome of the deme-deme conflict stage is visualised at the conclusion of, **a:** generation 20, **b:** generation 40, and **c:** generation 80.

**Determinant**		**Range**
Number of demes	m	$\mathbf{64}$
Effective deme size (one-third of census size)	n	$\mathbf{32}$
Average number of neighbours per individual	d	$4, \mathbf{6}, 8$
Within-deme fitness benefits of new technology	α	$1.2 - 4.0$
Periods per generation	T	$\mathbf{2000}$
Maximum coalition size for strategy updating	k	$\mathbf{2}$, 3, 4
Mistake rate in strategy updating	ε	$0.025, \mathbf{0.05}, 0.10$
Mutation rate from SI to N and vice versa	μ	$\mathbf{0.001}$
Per generation conflict probability	η	$0.05, \mathbf{0.10}, 0.20$

Table B: Parameter estimates. Benchmark values are in bold.

In Figure B the low-α regime is visualised. At the conclusion of generation 20 (Figure B **a**), four inter-deme contests are material, the demes being successfully invaded are indicated by the $\times$ symbol and the new, daughter, demes have been added to the top of the 'stack', and are encircled. In this case, since the new demes and the invaded demes exist at the same 'old' technology level for all contests A, B, C, D, the outcome of the contests have been decided by an equiprobable, random, choice as competing technologies would have been equal in all cases. At the conclusion of generation 20 we can also see several demes obtaining higher technology step levels of 2 and 3.

By generation 40 (Figure B **b**) a larger diversity of deme technology is apparent, with a degree of type-based superiority being established: only high N type fraction demes exist at technology step 4. Two material contests are apparent (A and B), both contests having been decided by technological superiority: both invaded demes sat at technology step 1 whilst the invading demes had advanced to technology step 3. Thus, the daughter demes, replacing the invaded demes, are created at technology step 3, a key part of the dynamics of technology and types in our model.

By generation 80 (Figure B **c**) almost all demes are dominated by N type individuals. Material inter-deme competition between technologically unbalanced demes is still possible (as evidenced by event A) but such events, likely between demes of similar SI or N type fraction, will not result in material changes to the overall population fraction of SI or N types.

Hence, in these three 'frames', we can see the emergence of technologically superior, N type dominated demes, which, over time, via inter-deme contests, ultimately greatly reduce the number of SI types in the population. Any emergence of SI types in a given deme due to drift, will be short-lived, as for α less than the threshold value for these parameters, demes with high numbers of SI types suffer from the conservative effect of shared intentions ($k > 1$) and will eventually fall behind the technology frontier.

In Figure C an above threshold scenario is visualised, with benchmark conditions and $\alpha = 2.2$ used. Again, we visualise a single replicate at generations 20, 40 and 80. In this case, generation 20 (Figure C **a**) reveals a wider range of technology in use across the demes owing to the higher rate of new technology adoption implicit in the higher α value. Nevertheless, already at the conclusion of generation 20 it can be seen that the incidence of demes dominated by N types is low, with higher technology steps more likely to be occupied by demes with mid to high numbers of SI types, leading, over time, to the eradication of demes with high numbers of N types.

By generation 40 (Figure C **b**), the dominance of SI types is entrenched, with the few remaining demes with high numbers of N types soon to be eradicated. By generation 80 (Figure C **c**) demes with few SI types are non-existent, with any remaining variation in type due to mutation and genetic drift.[3]

3 Choice of benchmark parameters

Here we explain the reasoning behind our choice of benchmark parameters. Our choice of $m = 64$ is both computationally manageable and also similar to the typical number of tribes found in a single Aboriginal language zone as determined by the Australian Institute of Aboriginal and Torres Strait Islander Studies (AIATSIS). Data from the AUSTLANG project (`http://austlang.aiatsis.gov.au/main.php`) shows

[3] It is interesting to note that the idea that differing speeds of norm adoption can lead to inter-demic selection implies that existing studies of the effects of different network configurations on the speed of norm adoption Centola and Baronchelli (2015); Kreindler and Young (2014); Montanari and Saberi (2010); Young (2011) could be used to further study the evolution of the human proclivity to form specific types of social networks.

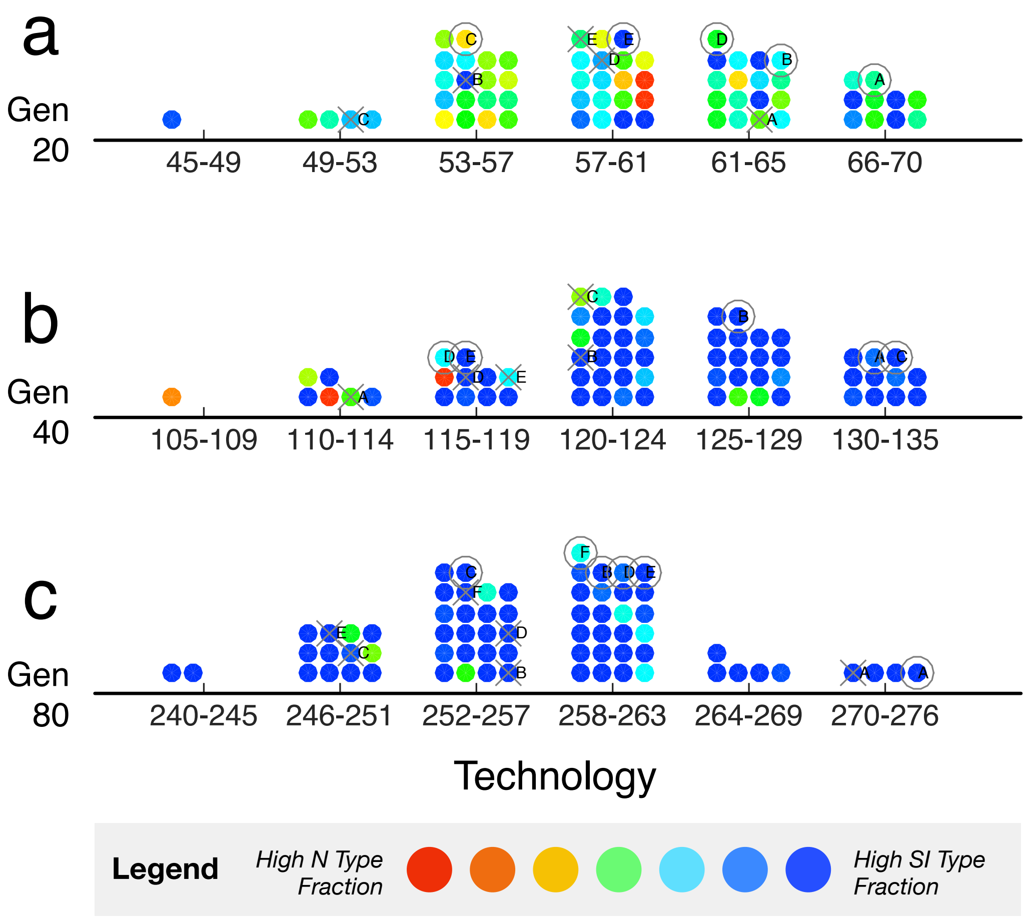

Figure C: **Example deme extinction and type evolution visualisation at benchmark parameter settings and $\alpha = 2.2$ (single replicate).** See caption to Figure **B** for details. Note: technology level scales are not consistent across panels.

that of 12 linguistic zones, 9 are listed as having a number of languages and dialects in the range 45-103. Our choice of $n = 32$ is similar to that in Bowles (2006) and is an estimate of the number of individuals who are able to breed in any given generation, that is approximately one third of census size. In addition, our choice is informed by the survey of Hill et al. (2011), who analysed 32 modern hunter-gatherer societies (total 5,067 individuals) and found that mean 'band' (adult members of a residential unit) was 28.2 (range 5.8 to 81.6).

d, which determines the number of edges in our scale-free graphs, as discussed in Section 4, was constrained by the computing power at our disposal, although it seems intuitively plausible that most individuals will be predominantly influenced by relatively few others (friends, family, hunting partners).

α is the focus of our treatments and is examined across a range of values that lead to both the evolution and non-evolution of SI.

Our choice of $T = 2000$ assumes that updating of actions is relatively rare, with the opportunity for some set of individuals to update arising every 3-4 days. We could have used larger values of T. What this would do is to exaggerate the technological differences between demes who are gaining technology fast and those who are gaining it slowly, increasing the selective effect at the inter-demic level.

We focus on small values of k because we wish to examine the evolution of collaboration and it is likely that the ability of pairs or small groups to share intentions would have to evolve prior to the ability of large groups to do likewise. Tomasello (2014) regards pairwise sharing of intentions as a special case as it only requires consideration of the first and second person (me and you) and not the third person (him). We agree with this reasoning and choose to make $k = 2$ our benchmark maximum coalition size.

The benchmark choice of $\varepsilon = 0.05$ was relatively arbitrary and corresponds to a mistake rate of one in twenty.

The mutation rate of $\mu = 0.001$ is high, but still only corresponds to an average of two mutations occuring in the metapopulation every generation. Working with lower mutation rates slows down initial waiting times until homogeneous populations become heterogeneous, but does not change the dynamics which occur thereafter, which is the object of interest in the current study.

Finally, the conflict probability η was chosen to be less than previous estimates. That is, we do not require unreasonably strong deme-level selection to get our results. This is further discussed in Section 2.4.

4 Social networks

4.1 Network type & Characteristics

Social networks play two important roles in the study. First, in each period individuals undertake pairwise productive activities with their neighbours, the product of their labours being determined by the coordination game in Table **A**. Second, up to k-vertex, connected subgraphs of SI types are able to jointly revise their strategy (see Figure 2 in the main paper). The same network informs both production and strategy revision. Unique social networks are generated afresh each generation for each deme, are always single-component and undirected, and are not altered during the generation.

We use 'scale-free' (SF) networks having an approximately power-law degree distribution (Barabasi and Albert, 1999). That is, the probability $P(d)$ of a vertex having d adjacent neighbours decays as a power law, $P(d) \sim d^{-\gamma}$. These networks are known as SF since for a certain range of γ, the average of the degree distribution does not converge – there is no 'characteristic' or 'expected' degree. In comparison to random networks where the function $P(d)$ decreases exponentially in d, the SF distribution exhibits so-called 'fat-tails': a much higher mass is located at large degrees than is the case with the Gaussian distribution.

SF networks have been discovered in many social, biological and physical systems (for a review, see Barabasi, 2009). Whilst it is impossible to identify the network structure of long-gone civilisations, recent, detailed study of the Hadza hunter-gatherers of Tanzania, a potentially representative Pleistocene-like culture, has demonstrated remarkable similarities between the social networks displayed within the Hadza and modern social network characteristics (Apicella et al., 2012). For instance, $P(d)$ was found to differ significantly from a random network distribution, with fat-tail phenomena present; ties were found to be strongly reciprocal (e.g. if A nominated B, implying $A \to B$ in g, then with high probability B nominated A, implying $B \to A$ in g, or simply $A \leftrightarrow B$ in g, note: nominations were private); and, ties were strongly

assortative – high in-degree vertices nominated more social contacts, whilst vertices with high out-degree were more likely to be nominated. Together, these features point to SF networks as being a reasonable analogue.

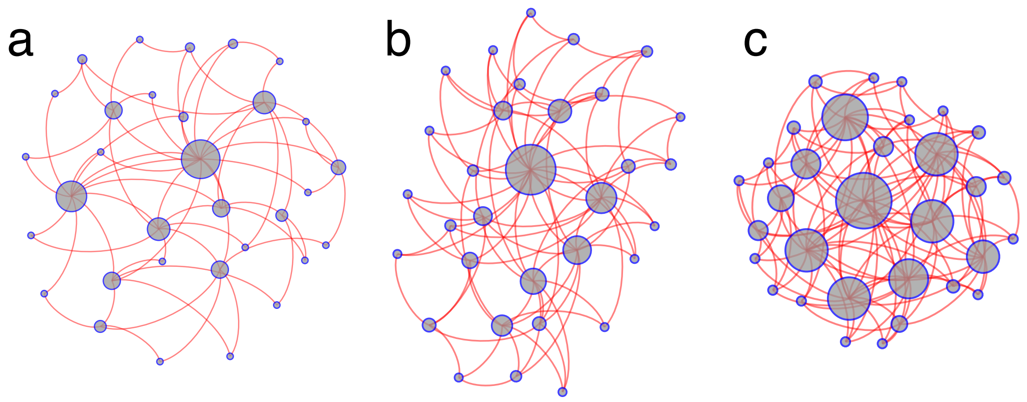

Figure D: **Example $n = 32$ scale-free networks used in the study. a:** Avg. degree $d \simeq 4$, **b:** $d \simeq 6$, and **c:** $d \simeq 8$.

To build the SF social networks, the Scale-Free Network Generator algorithm (Barabasi and Albert, 1999) was used as implemented in MATLAB by George (2007) and freely available online.[4] Example SF social networks of size $n = 32$ and approximate average degree 4, 6 and 8 are visualised in Figure **D**.

Table C: **Average network characteristics of social networks used in the study.** The benchmark case utilises $d \simeq 6$. The final column provides random networks of similar average degree to the benchmark for comparison.

Measure	Scale-free (study)						Random	
	$d \simeq 4$		$d \simeq 6$		$d \simeq 8$		$d \simeq 6$	
	mean	s.d.	mean	s.d	mean	s.d.	mean	s.d.
Degree								
mean	3.8	(0.094)	5.5	(0.12)	7.6	(0.47)	5.7	(0.12)
min	1.8	(0.43)	3.7	(0.49)	3.8	(0.603)	2.1	(0.81)
max	13.7	(2.4)	16.0	(2.2)	18.4	(2.0)	9.8	(1.1)
Clustering Coefficient	0.28	(0.090)	0.32	(0.061)	0.38	(0.047)	0.17	(0.031)
Characteristic Path Length	2.4	(0.078)	2.1	(0.037)	1.9	(0.044)	2.1	0.034
Diameter	4.3	(0.48)	3.7	(0.47)	3.0	(0.14)	4.0	(0.39)
$P(d)$, power-law exp, γ	2.50	(0.21)	2.65	(0.24)	2.73	(0.25)	-	-
Replicates	10,000		10,000		10,000		10,000	

Common, average charactersitics of the social networks used in the study are given in Table **C** after measuring each characteristics over 10,000 replicates of the network generator at each average degree level.[5] The last columns of the table include an equivalent ensemble characterisation of 10,000 random graphs (built by 100% random rewiring of a regular lattice) at approx. degree 6. Comparison of the the SF (study) ($d \simeq 6$) to the random graph data reveal expected higher clustering (0.32 to 0.17), equivalent path length (2.1 to 2.1) and diameter (3.7 to 4.0), and much larger average maximal degree (16.0 to 9.8) in the SF networks over the random graphs.

[4]See `http://bit.ly/1yabAKp` .

[5]Clustering coefficient, characteristic path length and diameter measures utilise the MIT Strategic Engineering lab's 'Matlab Tools for Network Analysis (2006-2011)' availble at `http://bit.ly/1AJrKt3`; exponent of the power-law distribution of vertex degrees follows Clauset et al. (2009) and utilises the accompanying MATLAB toolset avaiable at `http://bit.ly/1Gu4R0A`.

It is worth noting that whilst we believe the choice of SF social networks to be the most appropriate given considerations above, our previous work (Newton and Angus, 2013, 2015) demonstrates that conservative and reforming effects of $k > 1$ strategic revisions in social networks are observed in a wide range of network types including regular, 'small-world' and random versions of lattice networks, along with five empirical social networks drawn from a range of contemporary sources (n range 22 to 379).

4.2 Coalition formation

Strategy updating is either individual or proceeds by coalitional strategy updating in which a pair ($k = 2$) or more ($k > 2$) of SI type individuals, who are neighbours in the social network, can share intentions and develop a *coalitional better response*. Within each replicate of our study, each social network $g|_{n,d}$ is likely unique given the vast space of such networks, hence, for each deme, at generation initialisation, after the social network has been generated, a feasible coalitional database formation algorithm is run. The resultant database is stored in a specific data-structure which enables fast recall since the strategy updating step is called once every period (i.e. 128,000 times per generation) with, for example, around 50% of these calls for $k = 2$ being coalitional in nature.

For $k = 2$ the coalitional formation algorithm is straightforward: all edges in g between two SI types are viable coalitions and are added to the database.

For $k = 3$ the set of coalitions of size 2, is used as seed-coalitions, each seed-coalition being addressed in turn, adjacent SI type vertices being identified, and new size 3 coalitions being added to the database if not present already.

For larger k, the algorithm proceeds iteratively as for $k = 3$, building up from size 2, then 3, .. and so on, until the full coalitional set for the given k is found.

Naturally, for low SI fraction demes, the coalition database formation step is very fast. As SI fraction approaches 1.0, and $k \gg 2$ the algorithm can take several seconds, as the number of feasible coalitions becomes very large. However, for small k the algorithm is very efficient: up to $k = 3$ coalition formation with a 100% SI type, $n = 32$ SF network, plus 10,000 calls to the library takes $\sim$ 3s on a single core machine.

Run-time library lookup proceeds first by an equiprobable choice of coalition size up to k. For example for $k = 2$, approx. 50% of the time a single individual will be selected for strategy revision, and approx. 50% of the time, a $k = 2$ coalition will be considered, but only if the starting individual is of SI type. Even in this case, a 2 member coalition may not be feasible because the SI type starting individual has no SI type neighbours in g.

Computationally, an alternative approach to pre-construction of the feasible coalitional set for a given g and k would be to conduct run-time coalition formation, i.e. randomly select some individual, and build a coalition of up to size k including that individual (if SI type, and having SI neighbours). However, simulation testing indicated that pre-defining the universe of feasible coalitions of up to size k for a given g and run-time look-up was around 11 times faster than run-time coalition formation.

5 Additional Experiments: Robustness, the long run & placebo trials

5.1 Robustness

To explore the robustness of the study's main results to variations in the key parameters a full-factorial design survey of the convergence properties of the model under low ($\alpha = 1.2$) and high ($\alpha = 4.0$) rates of technological change was conducted. Four parameters were varied over a wide treatment set, given in Table D, leading to 81 experiments in all (see Table E for details).

In the low α treatments, individuals were randomly assigned to SI or N type with 0.50 probability at initiation, whereas in the high α treatments, experiments were initiated with a full-N type population, echoing the approach of the respective studies reported in the main paper. Each experiment was conducted over 20 unique random seeds (i.e. 1,620 model runs in all).

Since the focus of these experiments was to test whether the dynamics would *eradicate*, or *fully establish*, SI types in the population under low, or high, rates of technological change respectively, each replicate was stopped, with the generation number recorded, when the convergence criteria was met. Convergence

Table D: **Parameter values surveyed in the robustness study.** Benchmark values are underlined.

**Parameter**		**Treatment Set**
Maximum coalition size for strategy updating	k	$\{\underline{2}, 3, 4\}$
Average number of neighbours per individual	d	$\{4, \underline{6}, 8\}$
Per generation conflict probability	η	$\{0.05, \underline{0.10}, 0.20\}$
Mistake rate in strategy updating	ϵ	$\{0.025, \underline{0.05}, 0.10\}$

required more than 75% of the demes (i.e. at least 49 of 64 demes) to each exhibit at least 90% population fraction of N types (low α treatments) or SI types (high α treatments) respectively at the conclusion of the generation.

Table E: **Type convergence results of 81 full-factorial robustness experiments.** Reported results arise from 20 independent random seeds: **panel a**: 50% SI-type population intitial conditions, wait-time for each replicate to reach the 'no-SI' convergence threshold; **panel b**: 0% SI-type population initial conditions, wait-time for each replicate to reach the 'full-SI' convergence threshold. Benchmark conditions indicated by bold-face. * indicates largest wait-time (at $k = 2$) parameter combination.

Exp	k	d	η	ϵ	Convergence Wait-time (generations)					
					a			b		
					$\alpha = 1.2$			$\alpha = 4.0$		
					mean	s.d.	range	mean	s.d.	range
1	**2**	4	0.05	0.025	81	(18)	41 – 117	86	(10)	65 – 104
2	.	.	.	0.050	74	(16)	48 – 107	87	(14)	66 – 111
3	.	.	.	0.100	116	(26)	69 – 164	84	(13)	55 – 109
4	.	.	0.10	0.025	55	(10)	41 – 77	56	(7)	47 – 73
5	.	.	.	0.050	54	(8)	37 – 67	56	(8)	41 – 78
6	.	.	.	0.100	109	(56)	55 – 291	56	(8)	45 – 78
7	.	.	0.20	0.025	48	(14)	26 – 77	38	(7)	28 – 52
8	.	.	.	0.050	39	(6)	29 – 50	38	(8)	28 – 54
9	.	.	.	0.100	67	(28)	34 – 128	43	(7)	32 – 55
10	.	**6**	0.05	0.025	245	(128)	110 – 659	86	(14)	70 – 119
11	.	.	.	0.050	126	(31)	80 – 196	87	(11)	63 – 114
12	.	.	.	0.100	88	(17)	64 – 141	82	(7)	68 – 91
13	.	.	**0.10**	0.025	128	(44)	61 – 190	57	(7)	43 – 75
14	.	.	.	**0.050**	**78**	**(19)**	**51 – 122**	**56**	**(8)**	**40 – 71**
15	.	.	.	0.100	63	(16)	35 – 104	63	(9)	45 – 79
16	.	.	0.20	0.025	162	(165)	46 – 676	38	(6)	30 – 51
17	.	.	.	0.050	61	(20)	33 – 97	41	(6)	32 – 55
18	.	.	.	0.100	51	(16)	26 – 81	45	(11)	34 – 66
19*	.	8	0.05	0.025	425	(346)	98 – 1309	101	(11)	80 – 121
20	.	.	.	0.050	148	(47)	86 – 238	99	(16)	74 – 143
21	.	.	.	0.100	85	(14)	56 – 118	89	(12)	68 – 114
22	.	.	0.10	0.025	227	(138)	84 – 532	72	(15)	51 – 106
23	.	.	.	0.050	101	(38)	44 – 178	62	(12)	46 – 92
24	.	.	.	0.100	57	(11)	39 – 87	62	(8)	48 – 80
25	.	.	0.20	0.025	295	(299)	52 – 1342	54	(20)	40 – 127
26	.	.	.	0.050	78	(56)	31 – 287	44	(8)	33 – 57

(Table continues over page ...)

(... Continued from previous page)

Exp	k	d	η	ϵ	Convergence Wait-time (generations) a $\alpha = 1.2$ mean	s.d.	range	b $\alpha = 4.0$ mean	s.d.	range
27	.	.	.	0.100	45	(10)	33 – 63	46	(7)	35 – 67
28	3	4	0.05	0.025	75	(15)	51 – 122	76	(10)	63 – 101
29	.	.	.	0.050	71	(13)	52 – 93	78	(9)	66 – 96
30	.	.	.	0.100	83	(17)	59 – 122	81	(12)	52 – 106
31	.	.	0.10	0.025	54	(7)	43 – 64	53	(8)	37 – 71
32	.	.	.	0.050	46	(7)	32 – 60	56	(9)	44 – 72
33	.	.	.	0.100	56	(10)	36 – 77	54	(10)	41 – 80
34	.	.	0.20	0.025	39	(9)	24 – 54	34	(8)	25 – 51
35	.	.	.	0.050	31	(6)	19 – 45	35	(6)	28 – 56
36	.	.	.	0.100	43	(12)	22 – 71	39	(5)	31 – 49
37	.	6	0.05	0.025	244	(107)	135 – 562	79	(11)	59 – 98
38	.	.	.	0.050	107	(29)	62 – 163	79	(12)	63 – 102
39	.	.	.	0.100	79	(15)	57 – 112	84	(11)	68 – 107
40	.	.	0.10	0.025	208	(195)	55 – 695	55	(8)	43 – 70
41	.	.	.	0.050	91	(27)	54 – 135	50	(6)	41 – 62
42	.	.	.	0.100	59	(12)	36 – 84	54	(9)	40 – 75
43	.	.	0.20	0.025	145	(205)	32 – 981	39	(6)	29 – 51
44	.	.	.	0.050	68	(28)	25 – 150	37	(5)	29 – 52
45	.	.	.	0.100	43	(10)	27 – 67	35	(6)	28 – 46
46	.	8	0.05	0.025	412	(335)	110 – 1638	89	(18)	63 – 126
47	.	.	.	0.050	164	(54)	92 – 295	85	(12)	65 – 108
48	.	.	.	0.100	86	(14)	62 – 121	77	(12)	58 – 101
49	.	.	0.10	0.025	241	(219)	67 – 943	64	(16)	46 – 115
50	.	.	.	0.050	111	(84)	50 – 423	58	(12)	39 – 97
51	.	.	.	0.100	58	(10)	44 – 80	52	(7)	39 – 63
52	.	.	0.20	0.025	204	(232)	35 – 1099	44	(7)	29 – 54
53	.	.	.	0.050	65	(27)	34 – 146	42	(6)	33 – 56
54	.	.	.	0.100	42	(8)	31 – 58	38	(6)	25 – 51
55	4	4	0.05	0.025	72	(15)	56 – 117	79	(8)	56 – 95
56	.	.	.	0.050	71	(9)	56 – 88	74	(9)	62 – 90
57	.	.	.	0.100	82	(15)	57 – 108	80	(12)	52 – 95
58	.	.	0.10	0.025	46	(8)	30 – 67	51	(8)	42 – 71
59	.	.	.	0.050	47	(11)	30 – 76	50	(7)	40 – 64
60	.	.	.	0.100	49	(8)	36 – 70	52	(6)	40 – 64
61	.	.	0.20	0.025	34	(5)	27 – 46	37	(8)	25 – 53
62	.	.	.	0.050	35	(7)	24 – 53	35	(5)	28 – 48
63	.	.	.	0.100	39	(13)	27 – 72	36	(7)	28 – 53
64	.	6	0.05	0.025	181	(70)	75 – 360	81	(12)	50 – 95
65	.	.	.	0.050	133	(37)	84 – 205	78	(12)	51 – 100
66	.	.	.	0.100	82	(15)	58 – 108	77	(10)	57 – 94
67	.	.	0.10	0.025	123	(48)	58 – 197	53	(7)	40 – 68
68	.	.	.	0.050	80	(32)	47 – 177	49	(10)	36 – 72
69	.	.	.	0.100	54	(9)	41 – 78	55	(9)	39 – 77
70	.	.	0.20	0.025	133	(136)	51 – 518	36	(6)	26 – 47
71	.	.	.	0.050	55	(20)	32 – 95	36	(8)	24 – 55
72	.	.	.	0.100	41	(11)	24 – 78	33	(5)	24 – 46
73	.	8	0.05	0.025	393	(253)	143 – 1270	84	(12)	69 – 118
74	.	.	.	0.050	162	(88)	84 – 448	80	(13)	65 – 105

(Table continues over page ...)

(... Continued from previous page)

Exp	k	d	η	ϵ	Convergence Wait-time (generations)					
					a			b		
					$\alpha = 1.2$			$\alpha = 4.0$		
					mean	s.d.	range	mean	s.d.	range
75	.	.	.	0.100	82	(18)	51 – 122	76	(11)	59 – 103
76	.	.	0.10	0.025	309	(212)	61 – 690	61	(11)	44 – 85
77	.	.	.	0.050	107	(45)	49 – 202	58	(10)	41 – 76
78	.	.	.	0.100	62	(14)	42 – 89	52	(7)	42 – 64
79	.	.	0.20	0.025	298	(323)	43 – 1187	43	(12)	30 – 83
80	.	.	.	0.050	86	(69)	36 – 320	41	(9)	32 – 72
81	.	.	.	0.100	43	(9)	28 – 58	37	(6)	30 – 49

Convergence times (in generations) are given (mean, s.d., and range of 20 replicates) for all 81 treatments across both the low and high α settings in Table **E**. No replicate at any combination of parameters within the variable ranges specified failed to converge as defined within 2000 generations, with most replicates much faster.

5.2 The long run

Figure 4 in the main paper illustrates the mean final SI type population fraction after 500 generations across a range of α values with the system initialised to equiprobable SI or N type, at the benchmark parameter settings.

In Figure **E**, we repeat these simulations but allow them to run for a further 1,500 generations (i.e. to generation 2,000) to demonstrate the stability of the N and SI type regime under α of 1.2 and 2.2 respectively, which lie either side of the phase-transition point. To demonstrate the speed of SI type dominance under technology conditions above the transition point, the $\alpha = 2.2$ experiment (panel **b** in Figure **E**) was initiated with the SI type absent from all demes.

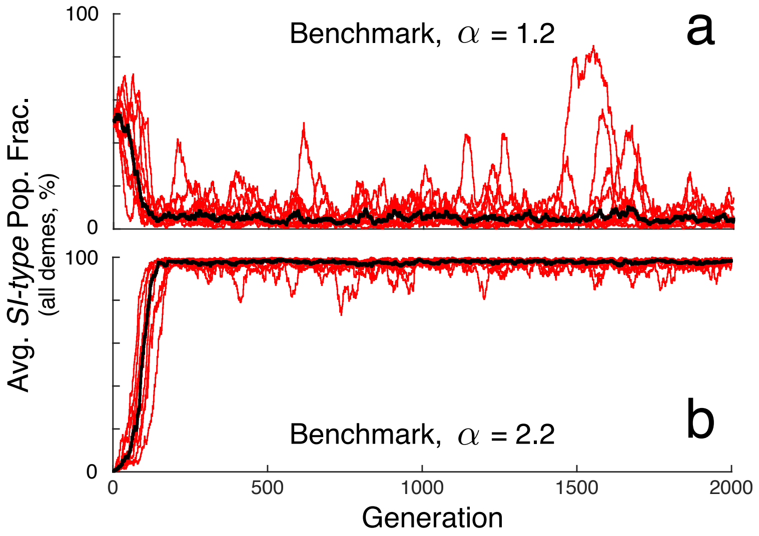

Figure E: **The stability of the N and SI type regimes for the benchmark case.** Red lines give the average population fraction (across all demes) of SI types for a single replicate (10 replicates are shown in all). Solid black line indicates the median of all replicates. In both panels, the benchmark parameter conditions are used. **a:** $\alpha = 1.2$ and initiation with equiprobable SI or N types across all demes, **b:** $\alpha = 2.2$ and initiation with SI types absent from all demes.

In Figure F we present long-run simulations of the slowest parameter combination identified in the robustness experiments for $k = 2$, namely, the $\{d = 8, \eta = 0.05, \varepsilon = 0.025\}$ experiment (refer Exp 19 in Table E). As with the robustness experiments, initiation saw 50% and 0% SI types in the starting population for the low $(\alpha = 1.2)$ and high $(\alpha = 4.0)$ regimes respectively.

Since very low conflict and strategy updating mistake-rate probabilities in the low α experiment give rise to very mild population selection dynamics, we run 40 replicates at the low α regime and present, in Figure F panel **a**, the 25th-75th percentile range, along with the median, across all replicates for clarity. We find that even under such weak population selection dynamics as given in this experiment, the N and SI type regimes are nevertheless remarkably stable over the long run within the low and high technology gradient respectively.

Given that the full-factorial robustness exercise demonstrated that all other parameter combinations imply stronger selection dynamics than this treatment (shorter wait-times), it would seem that the predictions of the N and SI type outcomes under low and high values of α respectively are robust to specific choices of the parameters within the wide ranges tested.

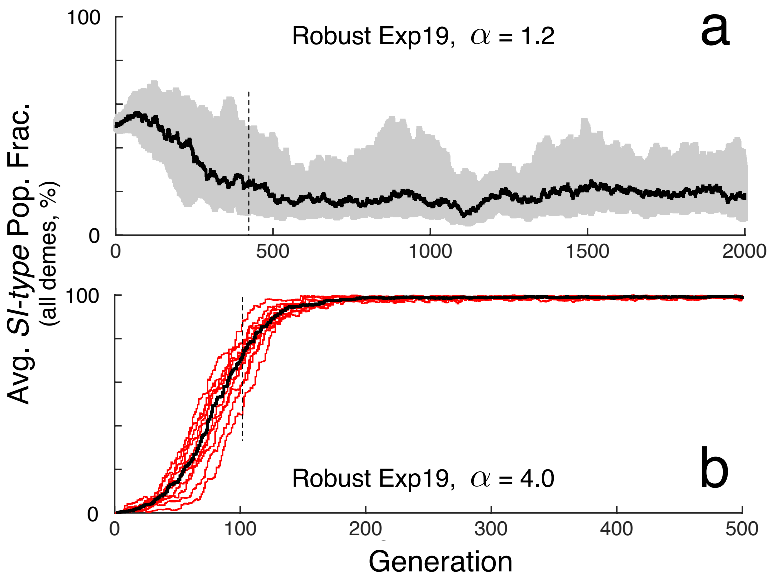

Figure F: **The stability of the N and SI type regimes under the longest wait-time parameter combination.** Average SI type fraction each generation across all demes from multiple replicates at the slowest wait-time parameter combination for $k = 2$ $(d = 8,\ \eta = 0.05,\ \varepsilon = 0.025$, refer Exp 19 in Table E). Dashed vertical lines in each panel give the mean convergence wait type (as reported in the table). **a:** The median (black line) and 25th to 75th percentile range (grey area) from 40 replicates is shown; **b:** The median (black line) and individual replicates (red line, 10 in all).

5.3 Placebo Trials

Here we report the results of placebo experiments to complement our main results. Placebo experiments serve two purposes: first, they provide one of the methods of model validation; and second, they provide information on the key drivers of the main results of a given model. We conduct placebo experiments under the benchmark parameter settings, for a below and above threshold α value over 500 generations, shadowing the trials reported in Figure 4 of the main study.

The two key stages of the model we enrol in the placebo experiments are the group and individual selection stages, represented by the *deme-deme conflict*, and *reproduction* stages respectively (refer Figure 1 of the main paper). Deme-deme conflict was switched 'on' or 'off' simply by entering the deme-deme conflict module or skipping it respectively. Alternatively, reproduction was switched from replicator dynamics (RD) as used in the study ('on'), to uniform probabilistic selection in the placebo 'off' setting.

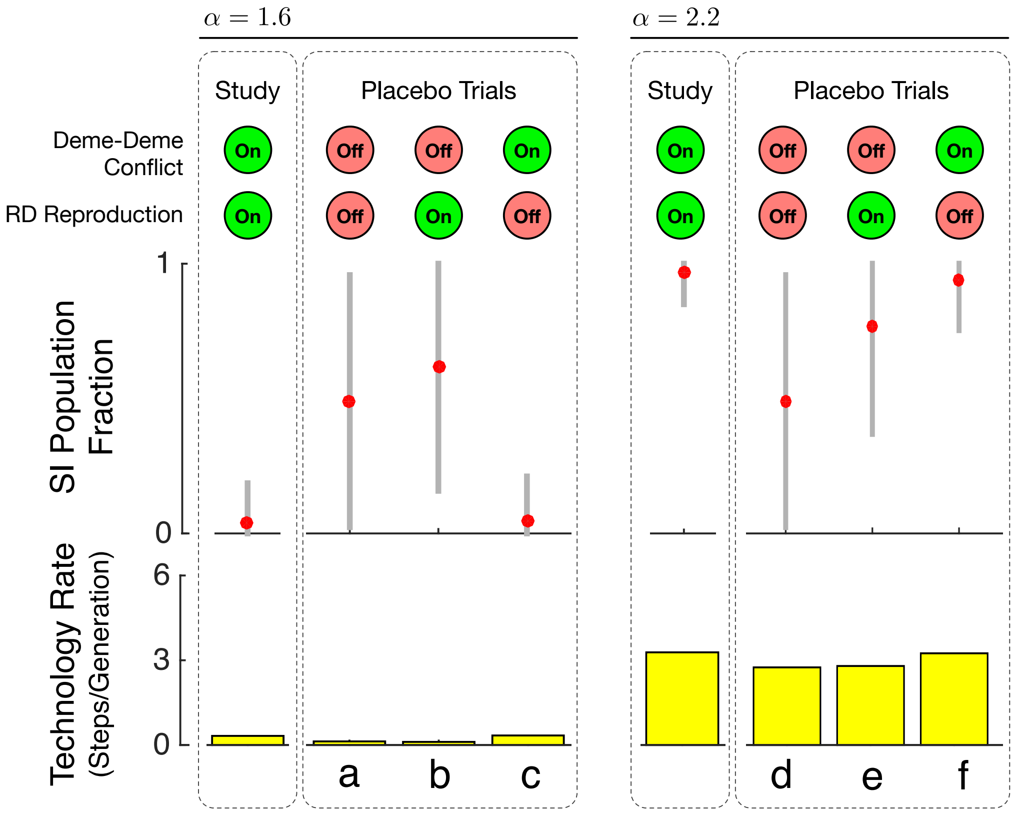

Figure G: **Placebo results at benchmark conditions and** $\alpha \in 1.6, 2.2$ **to complement Figure 4 in the main study.** Results obtained for SI population fraction (top panel) and Technology Rate (bottom panel) as per Figure 4 in the main study (i.e. mean results reported from generations 451 to 500 over 10 replicates). Placebo trials **a**, **b**, **d** and **e** skipped the Deme Extinction stage, while it was retained in **c** and **f**. Uniform probabilistic, rather than RD, reproduction was used in **a**, **c**, **d** and **f**.

In Figure **G** (left) we report the placebo results of a below-threshold treatment ($\alpha = 1.6$), complementing Figure 4 (Phase I) in the main study. As can be seen in the figure, both placebo trials where deme-deme conflict was skipped obtained no SI or N type dominance across the population, irrespective of the use of uniform probabilistic or RD reproduction (Figure **G** **a** and **b**). Whereas, when the Deme Extinction stage was used as per the main study (**c**), without RD reproduction, the study results were recovered.

In Figure **G** (right) we report the placebo results of an above-threshold treatment ($\alpha = 2.2$), complementing Figure 4 (Phase II) in the main study. Here, it is apparent that SI types can predominate in the metapopulation even without inter-demic selection. However, this dominance is further strengthened by inter-demic selection.

Next, we explore the robustness of the model's main results to two additional manipulations: i) inter-demic migration; and ii) less than 100% replacement of members of an invaded deme by replicas of members of a succesfully invading deme.

5.4 Migration

Amongst studies which explore the emergence of individual behavioural traits and include selection at an inter-demic level, *migration* between demes is sometimes considered. A recent survey of eight such papers finds that three of them consider migration but five do not (Table S1, Rusch, 2014). Migration of individuals amongst demes is of interest since any process which leads to the accumulation of substantial inter-demic diversity will be tempered or extinguished completely by inter-demic mixing (Bowles, 2006).

Migration is not part of our benchmark model. To explore the influence that migration might have, we add a random migration stage to the benchmark model. This takes place after the reproduction stage and prior to the next generation (refer Fig. 1 of the main paper). A single migration event was implemented as follows:

1. Choose two different demes at random, A and B;
2. Choose at random, individual i from deme A, and individual j from deme B;
3. Move individual i to deme B and individual j to deme A. Note that this only has an effect if i and j are of different types, in which case it is equivalent to changing the type of a chosen individual in each deme.

Since the migration process occurs at the end of each generation, no complications arise from within-generational concerns such as payoffs and social networks. At the beginning of the next generation, no distinction is made between new immigrants and the rest of the individuals in a deme.

We conduct experiments where $1/2 Mmn$ migration events occur each generation. M is a new parameter, the *migration probability*, and m, n are the number of demes and the number of individuals per deme as per the benchmark model. Note that the migration process is necessarily neutral with regards to deme size, and as such, causes the movement of two individuals (not one) per migration event. Consequently, to allow for comparison with related studies, we add the $1/2$ multiplier to obtain the number of migration events per generation.

In Figs. **H** & **I** we present the results of the benchmark model with migration added at low- and high-values of α. We provide in each figure the benchmark results for comparison. Selection against SI for low α holds for M up to 2.5% and breaks down for $M \geq 5\%$, which is approximately equivalent to full mixing every twenty generations (Fig. **H**). For the high α case, selection for SI withstands any level of migration considered (Fig. **I**).

The level of migration under which selection against SI types is retained (for low α) is not dissimilar to similar thresholds in related work. For example, in the study of García and van den Bergh (2011), where the benchmark scenario has no migration, a migration rate of 5% requires a dramatic increase in the benefit/cost ratio of the studied social dilemma game to ensure that 'altruists' are selected for.

5.5 'Brutality' in conflict and replacement

In the benchmark model, if a deme goes extinct it is replaced by a replica of the (usually higher technology) 'invading' deme. This can be thought of as the higher technology deme 'winning' a conflict scenario and

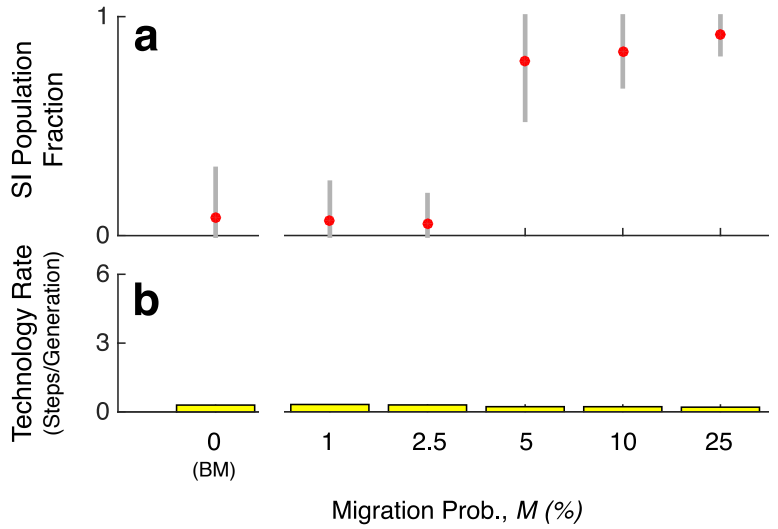

Figure H: **Robustness of selection against SI to migration at low alpha ($\alpha = 1.6$).** Results of **a**: SI population fraction; and **b**: technology rate over all 64 demes, 5 replicates and generations 451-500 mirror those of Fig. 4 in the main paper at benchmark conditions however with the migration module 'on' and migration probability M explored over five treatments as given (1% .. 25%). Benchmark (BM) results (no migration) given at left for comparison.

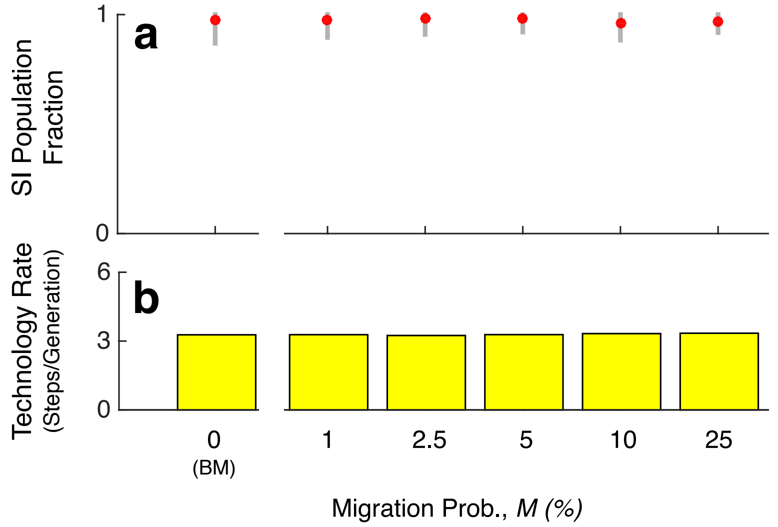

Figure I: **Robustness of selection for SI to migration at high alpha ($\alpha = 2.2$).** See caption to Fig. H for details.

effecting a policy of 100% 'brutality' on the loser (resulting in the complete replacement of the losing deme by the winning deme); or, one can think of the higher technology deme undergoing a fissioning process (resulting in the complete 'displacement' of the lower technology deme due to superior rates of productivity and associated fecundity).

Either interpretation is appropriate for our study, as the type of individuals only effects inter-demic selection indirectly through technological advance (see Section 2.4 for further discussion). Nevertheless, amongst related studies which consider a 'conflict' interpretation of inter-demic interaction, the fraction of the losing deme which is replaced by the winning deme – the level of *'brutality'* – is sometimes varied away from 100%.

Of the six relevant studies considered in Table S1 of Rusch (2014), only that of Choi and Bowles (2007) departs from 100% brutality. In the cited work, brutality is determined endogenously as a function of the relative strengths of the conflicting demes, with resultant average levels of brutality as high as 40% reported for some equilibrium states. In transient states, brutality levels of 0% to 100% are employed. However, comparison is not straightforward due to the far more elaborate deme-deme interaction scenario employed in the cited work (see Section 7 of the Supplementary Online Content of the cited paper for details).

In the spirit of Choi and Bowles (2007) we implement a less-than-unity 'brutality' experiment which respects the relative 'strengths' (technology steps) of the demes. Let the degree of replacement of the population of successfully invaded demes be given by β. We introduce parameter a to vary the level of β which applies when a deme is successfully invaded by another deme which has a technology level δ steps higher.

$$\beta(a, \delta) = 1 - 0.5^{a\delta+1} \ . \qquad (2)$$

In our benchmark setup, since a successfully invaded deme is replaced by a duplicate of the invading deme, all individual and group characteristics are copied over, including the technology step of the invading deme. In keeping with this approach, we set a floor of 50% on β, such that the new deme is populated with at least 50% of the individuals from the invading deme, with the remainder of the individuals coming from the invaded deme. This majority of individuals from the invading deme bring with them the invading deme's level of technology.

After every successful invasion, β is computed via (2), and a number (βn to the nearest integer) of individuals from the invading deme is chosen to replace the same number of randomly chosen individuals in the invaded deme. In both cases, random selection is conducted without replacement.

We note that this approach to brutality/displacement is inherently stochastic. Particularly, early in any given simulation the difference in the share of SI or N types between two demes is likely to be small, meaning that random selection is not at all guaranteed to lead to an increase in the type of individual that is represented in greater numbers by the invading deme. In this sense, whilst our floor of 50% brutality may appear high, the untargeted, stochastic, nature of selection implies a much weaker form of transmission from 'winner' to 'loser'. Further note that although we test reduced brutality/displacement, we do not correspondingly increase the probability η of such events occurring. That is, the rate of replacement of individuals by invaders during the Deme Extinction stage drops below its benchmark value of $\eta/2 = 5\%$.

Example evaluations of (2) are given in Fig. **J**. The specification implies that the brutality/displacement experienced by an invaded deme will be greater the more it lags behind the technology step of the invading deme. Parameter a enables control over the responsiveness of β to the difference in technology step, δ. Early in a simulation run it will typically be the case that $\delta = 0$ since demes start level in technology. Consequently, the level of displacement is set to the 50/50 floor. As the simulation progresses, technology differences may emerge, leading to $\delta > 0$ and consequently increasing β (when $a > 0$).

Again, the benchmark model was evaluated at the low- and high- α scenarios with a range of parameter values for $a \in 0.0, \ldots, 2.0$. Figures **K** and **L** give the results of each scenario respectively. Selection against SI at low α is returned for a range of treatments up to $a \leq 0.25$, whilst in the high α there is selection for SI for the full range of a explored.

To understand in more detail the way that (2) applies within the low α regime, we provide, in Fig. **M**, density plots of realised β for early and late generations across each treatment where $a > 0$. As expected, early in the simulation (**a**, top panel) a non-zero fraction of β realisations are at the $\beta = 0.5$ or 50/50 brutality/displacement level due to interacting demes having equal technology. However, as the simulation progresses (**b**, bottom panel), inter-demic differences in technology become the norm, with β densities

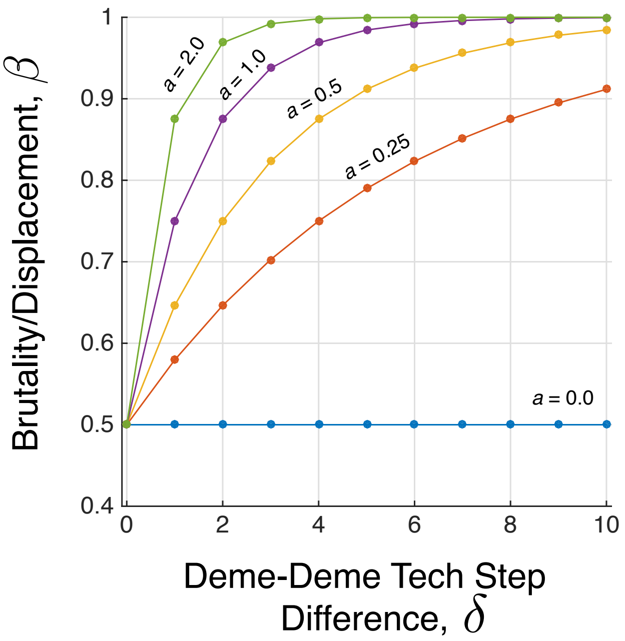

Figure J: **Example evaluations of 'brutality' equation** (2)**.** In the benchmark experiments, β is set to a constant of unity.

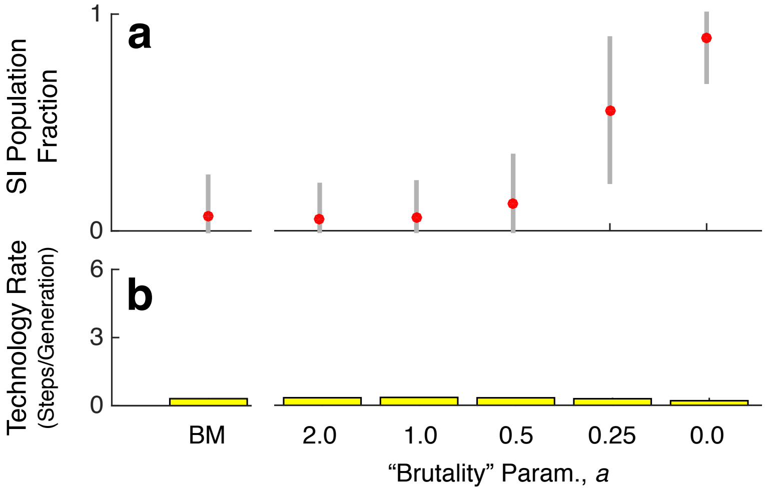

Avg. β	BM	2.0	1.0	0.5	0.25	0.0
Gen 1 to 50	1.00	0.85	0.80	0.71	0.63	0.50
Gen 451 to 500		0.91	0.86	0.77	0.69	

Figure K: **Robustness of selection against SI to 'brutality' $<$ 100% at low alpha ($\alpha = 1.6$).** Results of **a**: SI population fraction; and **b**: technology rate over all 64 demes, 5 replicates and generations 451-500 mirror those of Fig. 4 in the main paper at benchmark conditions however with β ('brutality') varied away from 100% by parameter a as indicated. Below the figure, average values of β are given over either the first-, or last-, 50 generations of the simulation run for $a > 0$. Benchmark results ($\beta = 1.00$) given at left for comparison.

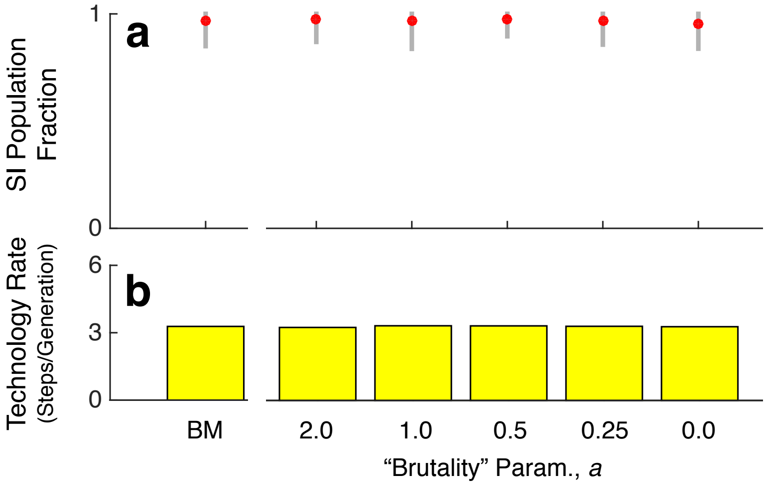

Avg. β						
Gen 1 to 50	1.00	0.94	0.90	0.84	0.75	0.50
Gen 451 to 500		0.97	0.95	0.91	0.83	

Figure L: **Robustness of selection for SI to 'brutality' $<$ 100% at high alpha ($\alpha = 2.2$).** See caption to Fig. K for details.

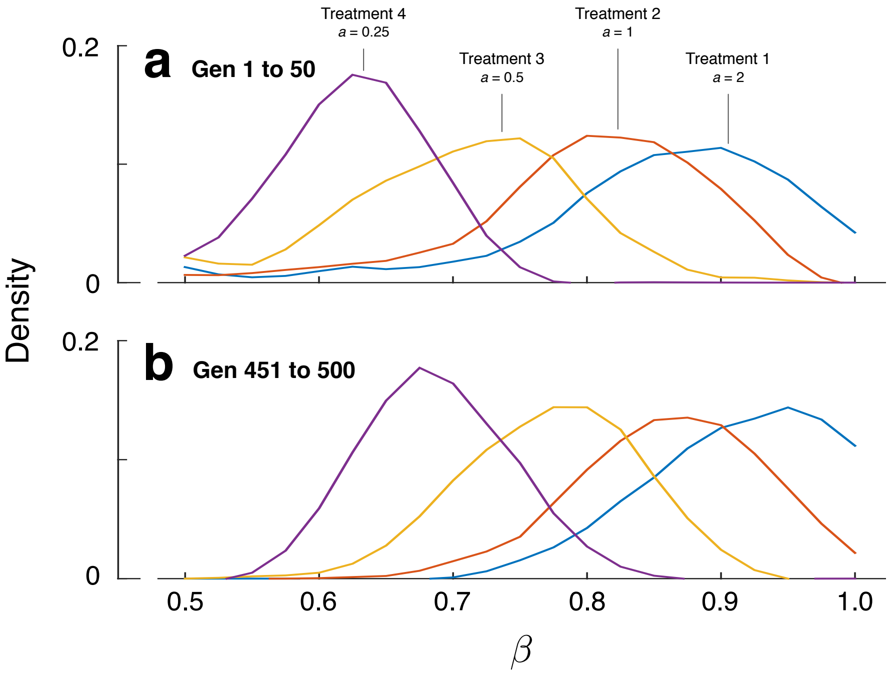

Figure M: **Resultant density of conflict brutality/displacement, β, in the low α experiments.** **a**: density of β across all inter-demic conflicts and generations 1 to 50, and **b**: the same for generations 451 to 500. Treatment numbers and values of a given in top panel with line colours retained in lower panel.

shifted to higher values. Note that even the $a = 0.5$ treatment, where the benchmark result for low α is retained, β values are drawn from a wide support on the unit interval, away from unity, even late in the simulation. Hence, we conclude that the model's main result in this regime is robust to considerable variance in the displacment level applied.

6 Numerical simulation

The model was implemented in the MATLAB programming language and run using one of three versions: R2013b, R2014a or R2014b as the project developed. The software were developed by the authors specifically for the project and all visualisations and data analysis was likewise conducted within the MATLAB environment. In addition to the main MATLAB primitive toolsets, the `parallel` and `statistics` toolboxes were used to parallelise computations (see below) and to conduct fitness-biased selection of parents during RD or uniform intra-deme reproduction. Post-processing of figures was achieved with OMNIGRAFFLE.

All simulations were conducted using the `parallel` toolbox with 8 to 24 threads on one of two MACPRO platforms (2010 or 2014 models). Parallelisation took place at the level of demes, i.e. sending a random selection of deme computations to be run in parallel for one generation. Any calling instance of MATLAB was run without graphical-user-interface from the Mac OS X Terminal or X11 xterm, typically over `ssh`.

Random number control was achieved by initiating each replicate with the replicate number as the seed to the MATLAB stream method `mt199937ar` (Mersenne Twister with Mersenne prime $2^{199937} - 1$). Stream initialisation ensured that any given replicate had identical, and reproducible, initial population conditions (initial individual SI or N type and technology choice, and assignment to a given deme). During generation initialisation, the network generator algorithm updated the stream with a unique, system clock-based seed, prior to network generation to ensure that our results did not depend in a sharp way on a particular sequence of networks being chosen from the vast global network space.

As an indication of run-times, utilising the 2014 MACPRO with 12 cores (12 GB RAM, SSD HD), a generation under benchmark conditions (2,000 periods, 64 demes of 32 individuals) took 4.3s, implying a single replicate of 500 generations (1×10^6 periods in all) as used, for example, in Figure 4 of the main paper, took approximately 36 min.

References

Alvard, M., 2001. Mutualistic hunting, in: Stanford, C., Bunn, H. (Eds.), The early human diet: The role of meat. Oxford University Press, Oxford, pp. 261–278.

Alvard, M.S., Nolin, D.A., 2002. Rousseau's whale hunt? Current Anthropology 43, 533–559.

Ambrus, A., 2009. Theories of coalitional rationality. Journal of Economic Theory 144, 676 – 695.

Apicella, C.L., Marlowe, F.W., Fowler, J.H., Christakis, N.A., 2012. Social networks and cooperation in hunter-gatherers. Nature 481, 497–501.

Aumann, R., 1959. Acceptable points in general cooperative n-person games, in: Tucker, A.W., Luce, R.D. (Eds.), Contributions to the Theory of Games IV. Princeton University Press, pp. 287–324.

Bacharach, M., 2006. Beyond individual choice: teams and frames in game theory. Princeton University Press.

Barabasi, A., Albert, R., 1999. Emergence of scaling in random networks. Science 286, 509–512.

Barabasi, A.L., 2009. Scale-Free Networks: A Decade and Beyond. Science 325, 412.

Baron-Cohen, S., 1994. From attention-goal psychology to belief-desire psychology, in: Baron-Cohen, S., Tager-Flusberg, H., Cohen, D.J. (Eds.), Understanding other minds: Perspectives from autism.. Oxford University Press.

Bernheim, B.D., Peleg, B., Whinston, M.D., 1987. Coalition-proof nash equilibria i. concepts. Journal of Economic Theory 42, 1–12.

Bird, R.B., Scelza, B., Bird, D.W., Smith, E.A., 2012. The hierarchy of virtue: mutualism, altruism and signaling in Martu women's cooperative hunting. Evolution and Human Behavior 33, 64–78.

Bowles, S., 2006. Group competition, reproductive leveling, and the evolution of human altruism. Science 314, 1569–1572.

Bratman, M.E., 1992. Shared cooperative activity. The Philosophical Review 101, 327–341.

Butterfill, S., 2012. Joint action and development. The Philosophical Quarterly 62, 23–47.

Call, J., 2009. Contrasting the social cognition of humans and nonhuman apes: The shared intentionality hypothesis. Topics in Cognitive Science 1, 368–379.

Call, J., Tomasello, M., 1999. A nonverbal false belief task: The performance of children and great apes. Child development 70, 381–395.

Carpenter, M., Akhtar, N., Tomasello, M., 1998a. Fourteen-through 18-month-old infants differentially imitate intentional and accidental actions. Infant Behavior and Development 21, 315–330.

Carpenter, M., Nagell, K., Tomasello, M., Butterworth, G., Moore, C., 1998b. Social cognition, joint attention, and communicative competence from 9 to 15 months of age. Monographs of the society for research in child development 63, 1–174.

Centola, D., Baronchelli, A., 2015. The spontaneous emergence of conventions: An experimental study of cultural evolution. Proceedings of the National Academy of Sciences 112, 1989–1994. http://www.pnas.org/content/112/7/1989.full.pdf.

Choi, J.K., Bowles, S., 2007. The coevolution of parochial altruism and war. Science 318, 636–640.

Clauset, A., Shalizi, C.R., Newman, M.E.J., 2009. Power-Law Distributions in Empirical Data. SIAM Review 51, 661–703.

Diamantoudi, E., Xue, L., Miyagawa, E., 2004. Random paths to stability in the roommate problem. Games and Economic Behavior 48, 18–28.

Eshel, I., Cavalli-Sforza, L.L., 1982. Assortment of encounters and evolution of cooperativeness. Proceedings of the National Academy of Sciences 79, 1331–1335.

Frayer, D.W., Martin, D.L., 2014. Troubled times: violence and warfare in the past. Routledge.

García, J., van den Bergh, J.C.J.M., 2011. Evolution of parochial altruism by multilevel selection. Evolution And Human Behavior 32, 277–287.

Gilbert, M., 1990. Walking together: A paradigmatic social phenomenon. Midwest Studies in Philosophy 15, 1–14.

Gold, N., Sugden, R., 2007. Collective intentions and team agency. The Journal of Philosophy 104, 109–137.

Hill, K.R., Hurtado, A.M., 1996. Ache life history: The ecology and demography of a foraging people. Transaction Publishers.

Hill, K.R., Walker, R.S., Božičević, M., Eder, J., Headland, T., Hewlett, B., Hurtado, A.M., Marlowe, F., Wiessner, P., Wood, B., 2011. Co-Residence Patterns in Hunter-Gatherer Societies Show Unique Human Social Structure. Science 331, 1286–1289.

Jackson, M.O., Watts, A., 2002. The evolution of social and economic networks. Journal of Economic Theory 106, 265–295.

Keeley, L.H., 1996. War before civilization. Oxford University Press.

Kelly, R.C., 2000. Warless societies and the origin of war. University of Michigan Press.

Klaus, B., Klijn, F., Walzl, M., 2010. Stochastic stability for roommate markets. Journal of Economic Theory 145, 2218 – 2240.

Konishi, H., Ray, D., 2003. Coalition formation as a dynamic process. Journal of Economic Theory 110, 1–41.

Kreindler, G.E., Young, H.P., 2014. Rapid innovation diffusion in social networks. Proceedings of the National Academy of Sciences 111, 10881–10888. http://www.pnas.org/content/111/Supplement_3/10881.full.pdf.

Luo, X., Yang, C.C., 2009. Bayesian coalitional rationalizability. Journal of Economic Theory 144, 248–263.

Moll, H., Tomasello, M., 2007. Cooperation and human cognition: the Vygotskian intelligence hypothesis. Philosophical Transactions of the Royal Society B: Biological Sciences 362, 639–648.

Montanari, A., Saberi, A., 2010. The spread of innovations in social networks. Proceedings of the National Academy of Sciences 107, 20196–20201.

Newton, J., 2012a. Coalitional stochastic stability. Games and Economic Behavior 75, 842–54.

Newton, J., 2012b. Recontracting and stochastic stability in cooperative games. Journal of Economic Theory 147, 364–81.

Newton, J., Angus, S., 2013. Coalitions, tipping points and the speed of evolution. University of Sydney Economics Working Paper Series 2013-02.

Newton, J., Angus, S.D., 2015. Coalitions, tipping points and the speed of evolution. Journal of Economic Theory 157, 172 – 187.

Newton, J., Sawa, R., 2015. A one-shot deviation principle for stability in matching problems. Journal of Economic Theory 157, 1 – 27.

Nowak, M.A., 2006. Five rules for the evolution of cooperation. Science 314, 1560–1563.

Peleg, B., Sudholter, P., 2003. Introduction to the theory of cooperative games, isbn 1-4020-7410-7 378 pages. Kluwer Academic, Boston .

Roth, A.E., Vande Vate, J.H., 1990. Random paths to stability in two-sided matching. Econometrica 58, 1475–80.

Rusch, H., 2014. The evolutionary interplay of intergroup conflict and altruism in humans: a review of parochial altruism theory and prospects for its extension. Proceedings of the Royal Society B: Biological Sciences 281, 20141539.

Sawa, R., 2014. Coalitional stochastic stability in games, networks and markets. Games and Economic Behavior 88, 90–111.

Searle, J., 1990. Collective intentions and actions, in: Cohen, P.R., Morgan, J., Pollack, M. (Eds.), Intentions in communication. MIT Press, pp. 401–15.

Searle, J.R., 1980. The intentionality of intention and action. Cognitive Science 4, 47–70.

Smith, E.A., 2003. Human cooperation: Perspectives from behavioral ecology, in: P.Hammerstein (Ed.), Genetic and cultural evolution of cooperation. MIT Press, pp. 401–427.

Sosis, R., Feldstein, S., Hill, K., 1998. Bargaining theory and cooperative fishing participation on Ifaluk atoll. Human Nature 9, 163–203.

Sterelny, K., 2014. Cooperation, culture, and conflict. The British Journal for the Philosophy of Science , axu024.

Tomasello, M., 2014. A natural history of human thinking. Harvard University Press.

Tomasello, M., Carpenter, M., 2007. Shared intentionality. Developmental science 10, 121–125.

Tomasello, M., Carpenter, M., Call, J., Behne, T., Moll, H., 2005. Understanding and sharing intentions: The origins of cultural cognition. Behavioral and brain sciences 28, 675–691.

Tomasello, M., Herrmann, E., 2010. Ape and human cognition what's the difference? Current Directions in Psychological Science 19, 3–8.

Tomasello, M., Rakoczy, H., 2003. What makes human cognition unique? from individual to shared to collective intentionality. Mind & Language 18, 121–147.

Traulsen, A., Nowak, M.A., 2006. Evolution of cooperation by multilevel selection. Proceedings of the National Academy of Sciences 103, 10952–10955.

Tuomela, R., Miller, K., 1988. We-intentions. Philosophical Studies 53, 367–389.

Velleman, J.D., 1997. How to share an intention. Philosophy and Phenomenological Research: A Quarterly Journal 57, 29–50.

Vygotsky, L.S., 1980. Mind in society: The development of higher psychological processes. Harvard University Press.

Wellman, H.M., Bartsch, K., 1994. Before belief: Childrens early psychological theory, in: Lewis, C., Mitchell, P. (Eds.), Childrens early understanding of mind: Origins and development. Hillsdale, NJ: LEA, pp. 331–354.

Wellman, H.M., Cross, D., Watson, J., 2001. Meta-analysis of theory-of-mind development: the truth about false belief. Child development 72, 655–684.

West, S.A., Griffin, A.S., Gardner, A., 2007. Social semantics: altruism, cooperation, mutualism, strong reciprocity and group selection. Journal of evolutionary biology 20, 415–432.

Wilson, D.S., Dugatkin, L.A., 1997. Group selection and assortative interactions. American Naturalist 149, 336–351.

Wobber, V., Herrmann, E., Hare, B., Wrangham, R., Tomasello, M., 2014. Differences in the early cognitive development of children and great apes. Developmental Psychobiology 56, 547–573.

Young, H.P., 1993. The evolution of conventions. Econometrica 61, 57–84.

Young, H.P., 2011. The dynamics of social innovation. Proceedings of the National Academy of Sciences 108 Suppl 4, 21285–91.
